# Fuzzy logic and Lyapunov‐based non‐linear controllers for HCV infection

**DOI:** 10.1049/syb2.12014

**Published:** 2021-03-29

**Authors:** Ali Hamza, Iftikhar Ahmad, Muhammad Uneeb

**Affiliations:** ^1^ Department of Electrical Engineering School of Electrical Engineering and Computer Science National University of Sciences and Technology (NUST) Islamabad Pakistan

## Abstract

Hepatitis C is the liver disease caused by the Hepatitis C virus (HCV) which can lead to serious health problems such as liver cancer. In this research work, the non‐linear model of HCV having three state variables (uninfected hepatocytes, infected hepatocytes and virions) and two control inputs has been taken into account, and four non‐linear controllers namely non‐linear PID controller, Lyapunov Redesign controller, Synergetic controller and Fuzzy Logic‐Based controller have been proposed to control HCV infection inside the human body. The controllers have been designed for the anti‐viral therapy in order to control the amount of uninfected hepatocytes to the desired safe limit and to track the amount of infected hepatocytes and virions to their reference value which is zero. One control input is the Pegylated interferon (peg‐IFN‐α) which acts in reducing the infected hepatocytes and the other input is ribavirin which blocks the production of virions. By doing so, the uninfected hepatocytes increase and achieve the required safe limit. Lyapunov stability analysis has been used to prove the stability of the whole system. The comparative analysis of the proposed nonlinear controllers using MATLAB/Simulink have been done with each other and with linear PID. These results depict that the infected hepatocytes and virions are reduced to the desired level, enhancing the rate of sustained virologic response (SVR) and reducing the treatment period as compared with previous strategies introduced in the literature.

## 1 INTRODUCTION

Viral diseases are the major cause of human morbidity and mortality around the world: HCV is one of the major contributor in this regard. It is a viral infectious disease of the liver which was first identified in 1989. Hepatitis A Virus (HAV) and Hepatitis B Virus (HBV) were discovered through the serological tests during 1980s. It was revealed that all transfusion‐associated Hepatitis cases were neither HAV nor HBV, therefore they were named as Non‐A, Non‐B Hepatitis (NANBH). Later , this specific viral genome was named as HCV [1]. It belongs to the Flaviviridae family and the Hepacivirus genus which is a positive‐strand ribonucleic acid (RNA) virus. The HCV RNA performs important tasks of holding its genetic information and data to make proteins for replication of the virus, both from its own part and the host liver cell. It is surrounded by layers of proteins and lipids carrying viral glycoproteins E1 and E2 which produce viral particles [2]. It is estimated that 25% of hepatocellular carcinoma (HCC) and 27% of cirrhosis have occurred due to the HCV [3]. According to the World Health Organization (WHO) report on HCV published in [4], approximately 71 million people are suffering from chronic HCV worldwide and about 1.75 million people are infected annually from which 3,99,000 patients have died and 8,43,000 have been recovered completely. High density of HCV infections has been observed in the Middle East/North Africa (MENA). Anti‐HCV prevalence rate is about 1.80% in Europe, 2.80% in Asia, 2.90% in Africa, 1.80% in Australia and 2.70% in MENA [5].

Anti‐viral therapy was used in the past to treat chronically infected patients due to the unavailability of the vaccine. The main purpose of the treatment of HCV is to avoid the chronic stages which include HCC, cirrhosis and liver cancer. Pegylated interferon, commonly called peg‐interferon, is a standard chemical‐modified form of interferon that treats Hepatitis C. Interferon alpha (IFN‐*α*) had been used for several years as an anti‐viral therapy for HCV treatment [6]. Other forms of treatment include combination therapy including peg‐IFN‐*α* with ribavirin, or the use of new Direct‐acting Antivirals Agents (DAAS) [7, 8]. IFN‐*α* is a protein synthesised by the immune system which fights against cancer and other diseases. It is made up of white blood cells present inside the human body and can also be produced artificially in laboratories to prepare medicines. It also communicates with the immune system to counter any attack of microorganisms (viral or bacterial infection). Three types of peg‐IFN‐*α* are available for the treatment: alfa (IFN‐*α*), beta (IFN‐*β*) and gamma (IFN‐*γ*) [9]. Ribavirin is a chemically formed nucleoside analogue of the ribofuranose which can be used for the treatment of deoxyribonucleic acid (DNA) and RNA diseases [10]. It is used with peg‐IFN‐*α* for chronic HCV treatment and increases the rate of SVR. SVR observes a virologic curve [11]. As the liver disease progresses over a long time, SVR test is prescribed to check the detection of any HCV viral particle which exits in the blood of the infected person.

After the discovery of HCV virus in late 20^*th*^ century, researchers started their efforts to analyse its viral kinetic modelling. First, a mathematical model was proposed by Neumann in [12] which was adopted from HBV [13] and HIV infections [14]. This model analysed the treatment of HCV only in the presence of interferon (IFN). It blocked the rate of production of virions from the infected hepatocytes, although it had very little impact on the infection of healthy cells. Dixit et al [15] improved the impact of peg‐IFN‐*α* by including the effect of ribavirin in [12]. This model incorporated biphasic decline pattern of HCV which showed that peg‐IFN‐*α* had a pivotal role during the first phase of declining the virions. During second phase, ribavirin had a significant contribution in the presence of low peg‐IFN‐*α* efficacy. Dahari et al [16] proposed a new model which included triphasic declining pattern of viral load. Recently, new dynamical models for HCV have been proposed under DAAS [17, 18, 19, 20, 21]. By considering the role ofthe immune system, several studies have been conducted to develop new models for stimulating therapeutic cells which reduce the viral load [22, 23, 24, 25].

Control theory has found a wide range of applications in biological and ecological problems [26]. Non‐linear control techniques play an extraordinary role in the biomedical field[27, 28, 29]. A fuzzy logic‐based optimal control has been proposed for solving the optimal control problem of HCV in [30]. Chakrabarty and Josh formulated an optimal function for HCV dynamics in [31] to decrease the viral load by using peg‐IFN‐*α* and ribavirin. By considering clinical trials, an optimal function was proposed to determine the optimal efficiency of the combined peg‐IFN‐*α* and ribavirin treatment of HCV [32]. An optimal treatment programme for HCV was considered spanning over a 10‐year period in [33] by considering the chronic infected, susceptible and treated injecting drug users. This work was based on offline optimal control method and was not affected by viral load management. Moreover, controller design was based on the nominal method and the limitation of control input was not considered. In the case of HCV treatment, the limitation of drug efficacy should be considered. Adaptive non‐linear controller has been proposed in [34] for the control of HBV infection. Practical limitations of treatment implementation, such as unavailability of states and efficacy limitations have not been considered in this work. Adaptive backstepping controller has been proposed in [35] where the limitation of the drug efficacy is taken into account. This work is carried out on the basic Neumann model [12] for HCV. The proposed control strategy lacked proliferation rate of infected and uninfected hepatocytes. Furthermore, only IFN‐based treatment strategy has been utilised.

In this article, an extended model of HCV proposed in [16] having the combination of peg‐IFN‐*α* and ribavirin as control inputs and non‐linear PID controller, Synergetic controller, Lyapunov Redesign controller and Fuzzy logic‐based controllers have been proposed. Drug efficacy limitation has been considered in the proposed strategy. This strategy enhances the rate of SVR and reduces the treatment period of the disease using minimum drug quantity as compared with strategies proposed in the literature. The peg‐IFN‐*α* is used for reducing infected hepatocytes and ribavirin is used for blocking virions for chronic infected patients. The objective of these controllers is to reduce and block infected hepatocytes and virions to their desired reference value which is zero. As a result, uninfected hepatocytes will be increased to the safe limit. All the proposed controllers have been compared with each other and with the linear PID, based on characteristics like steady state error (SSE), overshoots/undershoots, ripples/oscillations, transient time and settling time in the simulation results which are defined as:

- Steady state error: SSE is the difference between the actual output and the desired output at the infinite range of time.
- Overshoots: It is a straight way difference between the magnitude of the highest peak of time response and magnitude of its steady state.
- Ripples/oscillations: It is the repetitive variation, typically in time, of some measure about a central value or between two or more different states.
- Transient time: The time taken by the system to change from one steady state to another steady state is called the transient time.
- Settling time: The time required for a response to become steady. It is the time required by the response to reach and steady within specified range of 2% to 5% of its final value.

The Lyapunov stability theorem has been used to establish stability of the closed‐loop disease control.

This article has been organised as follows: non‐linear mathematical model of HCV has been explained in Section‐II. Treatment strategy and control objective is presented in section‐III. Design methodology for proposed non‐linear controllers has been given in section‐IV. Control design procedures for linear PID controller in IV‐A, non‐linear PID in IV‐B, Synergetic controller in IV‐C, Lyapunov Redesign controller in IV‐D and FLBC has been described in IV‐E. Simulation results, comparative analysis of the proposed non‐linear controllers with each other and with PID controllers, and performance of the proposed controllers under noise measurement have been demonstrated in section‐V. Section‐VI contains the conclusion. A list of References is given at the end of this article.

## 2 NON‐ LINEAR MATHEMATICAL MODEL OF HCV

Mathematical modelling allows inclusion of all relevant variables giving insight of the progression of the disease and the effects of treatment. First, a mathematical model was proposed by Neumann et al. [12] which was adopted from the model of HBV and HIV and the derived equations of HCV are given below:

(1) $$\left\{\begin{array}{cc} \frac{dT}{dt} & =\;s-dT-\beta VT\\ \frac{dI}{dt} & =\;\beta VT-\delta I\\ \frac{dV}{dt} & =\;\left(1-u\right)pI-cV \end{array}\right.$$

where *T* represents healthy cells, *I* means infected cells and *V* represents viral load. The parameters *s*, *d* and *β* are production rate, death rate and infection rate of healthy cells, respectively. Similarly, infected cells reduce at the rate of *δ*, and the virus produce at the rate of *p* and vanish at a constant rate of *c* per virus. Further, *u* is control input in the treatment strategy which is used as an anti‐viral drug for decreasing the production of the viral load by factor (1 − *u*). Drug efficacy *u* remains zero before and during treatment, its value should remain between 0 and 1.

Dahari analysed [12] and showed that this model considers the source of hepatocytes, completely ignores the proliferation of uninfected and infected hepatocytes and does not show any triphasic behaviour of the viral load. Further, Neumann model for chronic HCV assumed no proliferation of uninfected and infected hepatocytes (*r* = 0) and showed biphasic decline of viral load under therapy which consisted of first phase of rapid decline of viral load followed by shoulder phase in which viral load decayed slowly. Therefore, Dahari proposed a new mathematical model for chronic HCV by extending the Neumann model with the addition of the proliferation term for both uninfected and infected hepatocytes in [16].

A three state non‐linear mathematical model of HCV suggested by Dahari in [16] is considered for describing the dynamics of HCV infection for the combination of peg‐IFN‐*α* and the ribavirin therapy which shows a triphasic behaviour of the viral load where virions show sudden decay in the first phase of 1–4 days followed by the second phase (shoulder phase) of 20–30 days in which the virions decrease slowly and are eliminated completely at the end of the final phase. This model includes an extra term for the replication of uninfected and infected hepatocytes given by the following non‐linear firs‐ order system of differential equations:

(2) $$\left\{\begin{array}{cc} \frac{dT}{dt} & =\;s+r_{T}T\left(1-\frac{T+I}{T_{max}}\right)-d_{T}T-\left(1-\eta \right)\beta VT\\ \frac{dI}{dt} & =\;\left(1-\eta \right)\beta VT+r_{i}I\left(1-\frac{T+I}{T_{max}}\right)-\delta I\\ \frac{dV}{dt} & =\;\left(1-\epsilon_{p}\right)pI-cV \end{array}\right.$$

where *T* and *I* represent the state of the uninfected hepatocytes and the infected hepatocytes which can proliferate at the rate of *r*_*T*_ and *r*_*i*_, respectively. The maximum proliferation rate for the infected and uninfected hepatocytes can be differenconcentration of virions. The other model parameters *s*, *d*_*T*_, *σ*, *β*, *p* and *c* along their definitions are given in Table 1.

**TABLE 1:** Parameter definition

Variable	Parameter Definition	Unit
*S*	Rate of production of new uninfected hepatocytes	*cell mL* ^−1^ *day* ^−1^
*P*	Production rate of virions per cell	virions *day* ^−1^
*d*_*T*_	Death rate of uninfected hepatocytes	*day* ^−1^
*Β*	Rate of infection of new uninfected hepatocytes	*virions* ^−1^ *ml day* ^−1^
*C*	virion clearance rate	*day* ^−1^
*Δ*	infected hepatocytes death rate	*day* ^−1^
*r*_*T*_	uninfected hepatocytes proliferation rate	*day* ^−1^
*r*_*i*_	infected hepatocytes proliferation rate	*day* ^−1^

*T*_max_ is the concentration of the maximum uninfected hepatocytes inside human body and its value is 8,000,000 IU/ml. Proliferation rates allow liver growth until *T*_max_ is achieved. *T*_max_ is supposed to be the maximum uninfected hepatocytes and for that, we need to make sure two conditions which are:

$$s\;\leq d_{T}\;T_{max}\;\;\;\;T_{max}>\frac{s}{d}$$

**FIGURE 1:**
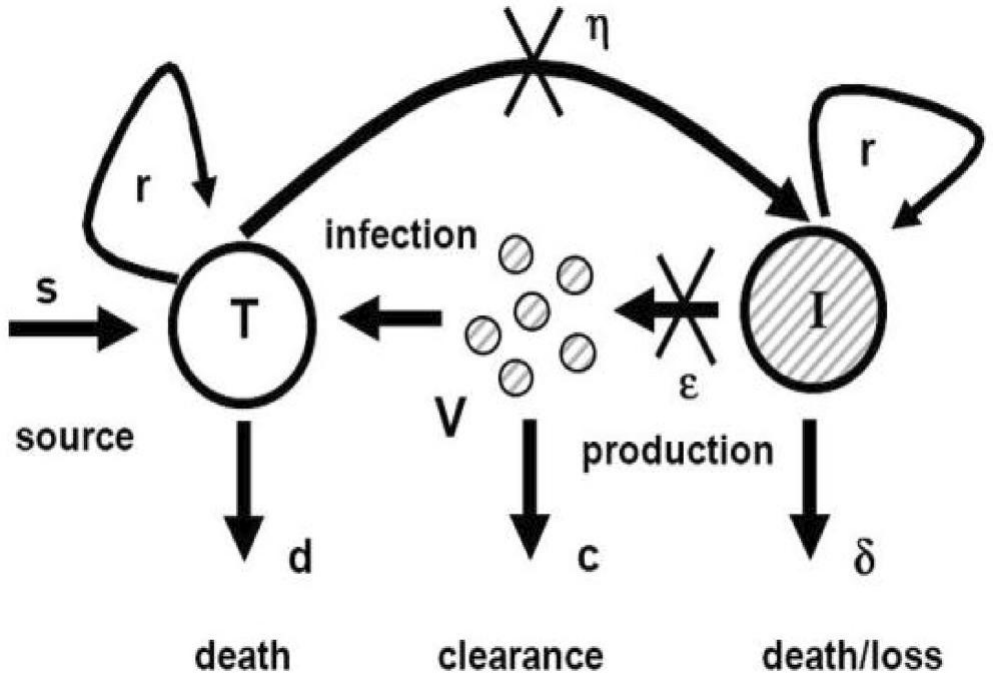
**Model of chronic HCV** [16]

Diagrammatic representation of chronic HCV with proliferation rates is shown in Figure 1.

It is convenient to change the variables from *T*, *I*, *V*, *η* and ε_*p*_ to *x*_1_, *x*_2_, *x*_3_, *u*_1_ and *u*_2_, respectively. The dynamics of HCV in eq. (2) become:

(3) $$\left\{\begin{array}{cc} \frac{dx_{1}}{dt} & =\;s+r_{T}\;x_{1}\;\left(1-\frac{x_{1}+x_{2}}{T_{max}}\right)-d_{T}\;x_{1}-\left(1-u_{1}\right)\beta \;x_{3}\;x_{1}\\ \frac{dx_{2}}{dt} & =\;\left(1-u_{1}\right)\beta \;x_{3}\;x_{1}+r_{i}\;x_{2}\;\left(1-\frac{x_{1}\;+\;x_{2}}{T_{max}}\right)-\delta \;x_{2}\\ \frac{dx_{3}}{dt} & =\;\left(1-u_{2}\right)\;p\;x_{2}-\;c\;x_{3} \end{array}\right.$$

The chronic HCV is treated with peg‐IFN‐*α* by the combination of ribavirin. Peg‐IFN‐*α* primarily functions by blocking virion production and allows the treatment of the de novo infection. The control inputs parameters *u*_1_ and *u*_2_ are the efficacy of the treatment used for the reduction of infected hepatocytes and blockage of production of virions inside the patient's body by the factors (1 − *u*_1_) and (1 − *u*_2_), respectively. Limitation of drug efficacy has been taken into account in order to make treatment realistic. The values of the control inputs *u*_1_ and *u*_2_ should remain between 0 and 1. Drug efficacy is limited and is given as:

$$control\;\;\;inputs=sat\left(u_{1},\;\;u_{2}\right)=\left\{\begin{array}{c} 0\leq u_{1}\leq 1\;\\ 0\leq u_{2}\leq 1.\; \end{array}\right.$$

In order to check the accuracy of this model, Snoeck [36] compared the clinical data of HCV with the predicted values obtained from the model and found that it has reasonable predictive capability. Also, this model has been approved by utilising clinical data of patients under combination of peg‐IFN‐*α* and ribavirin therapy.

## 3 CONTROL OBJECTIVE AND TREATMENT STRATEGY

In this article, the treatment strategy for the HCV disease using two control inputs, peg‐IFN‐*α* as *u*_1_ and ribavirin as *u*_2_ has been proposed which are based on the non‐linear PID, FLBC, Lyapunov Redesign and Synergetic controllers in such way that the following objectives can be achieved:

- Reduction and blockage of infected hepatocytes and virions to their desired reference value under treatment of control input laws within 8 to 12 weeks.
- Increasing uninfected hepatocytes to the maximum safe limit *T*_max_.
- System should be asymptotically stable globally.

The reference level is based on assumption that the infected hepatocytes and virions are completely eliminated and cleared from the human blood. The treatment strategy is based on the blockage and the reduction of the infected hepatocytes and virions using peg‐IFN‐*α* and ribavirin as control inputs within 8‐12 weeks. The treatment stops after achieving the objectives and the patient's blood samples are tested over the next 12 weeks, and if no viral load is detected, it is called sustained viroligic response (SVR) and the patient is cured completely. When SVR is achieved, it means that no viral load is detected inside blood and patient is HCV cured.

## 4 CONTROLLER DESIGN METHODOLOGY

**FIGURE 2:**
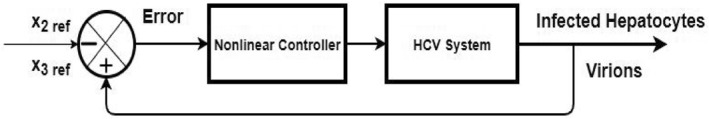
Closed loop control of HCV system

The HCV system eq. (2) is a non‐linear system due to the product of states *x*_3_ and *x*_1_. Hence, designing the non‐linear controller could help better in obtaining the desired control objectives of the system. The general representation of closed loop control system is shown in Figure 2 in which the states of the virions and the infected hepatocytes have been used as a feedback for the controller design to track their respective reference levels. Error equation has been introduced in the system by taking difference between states and their reference values.

Four non‐linear controllers―non‐linear PID controller, Synergetic controller, Lyapunov Redesign controller and FLBC―have been proposed in order to block and eliminate the infected hepatocytes and virions to their reference value by using antiviral drugs peg‐IFN‐*α* and ribavirin as control inputs. By doing so, uninfected hepatocytes converge to safe limit *T*_max_.

### 4.1 Linear PID controller

The PID control is a closed loop feedback control system widely used in various industrial automations due to its adaptability and reliability [37]. Its output *u*(*t*) has been calculated from feedback error in time domain as follows:

(4) $$u\left(t\right)=K_{p}e\left(t\right)+K_{i}\int e\left(t\right)dt+K_{d}\frac{de}{dt}$$

**FIGURE 3:**
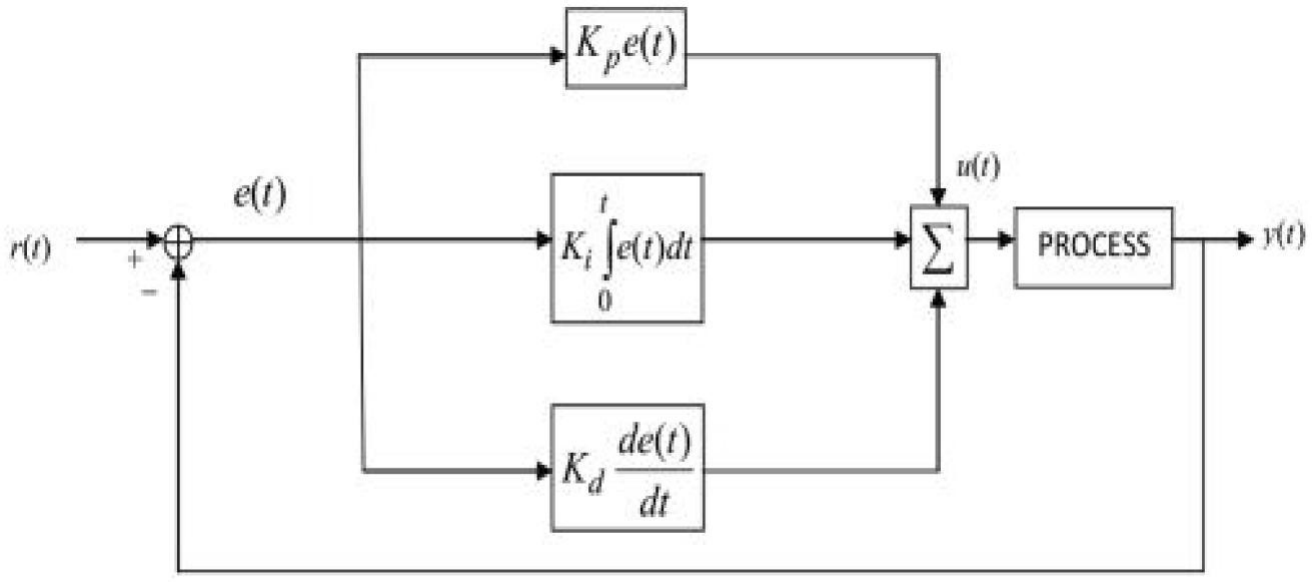
Block diagram of PID Controller

Error signal *e*(*t*) which is the difference between system output *y*(*t*) and desired reference level *r*(*t*) is fed into controller which determines the integral, derivative and proportional of error signal *e*(*t*) w.r.t. time. The controller output *u*(*t*) consists of a proportional gain *K*_*p*_ multiplied by the error *e*(*t*) plus integral gain *K*_*i*_ multiplied by the integral of *e*(*t*) plus the derivative gain *K*_*d*_ multiplied by the derivative of *e*(*t*) w.r.t. time. *u*(*t*) is then fed to the process to get the desired response by tuning these gains [37]. The block diagram of the working PID is shown in Figure 3.

### 4.2 Non‐linear PID controller

The non‐linear PID has the same structure as that of linear PID controller mentioned in eq. (4). However, gain and integral time of controller are not fixed. They are the non‐linear functions of the control error *e*. The non‐linear PID control algorithm [38], similar to eq. (4) is defined by:

(5) $$u_{i}\left(t\right)\;=\;K_{p}\;e_{i}\left[e_{i}\left(t\right)\;+\;\frac{1}{T_{i}\left(e_{i}\right)}\;\int_{0}^{t}\;e_{i}\left(t\right)d\left(t\right)\;+\;T_{d}\;\frac{d\;e_{i}\left(t\right)}{dt}\right]$$

where *K*_*p*_(*e*_*i*_) and *T*_*i*_(*e*_*i*_) are non‐linear controller gain and integral time as function of control error *e*_*i*_, respectively, defined as:

(6) $$K_{p}\;=\;K_{max}\;-\;\left(K_{max}\;-\;K_{min}\right)\;\left(1\;+\;a_{p}\;|e|\right)exp\;\left(-a_{p}\;|e|\right)$$

where *K*_max_ > *K*_min_. *K*_max_, *K*_min_ are the maximum and minimum values of the controller gain. *K*_*p*_ is bounded by *K*_max_ ≥ *K*_*p*_ ≥ *K*_min_ and *a*_*p*_ is positive parameter.

Similarly,

(7) $$T_{i}\;=\;T_{max}\;-\;\left(T_{max}\;-\;T_{min}\right)\;\left(1\;+\;a_{i}\;|e|\right)exp\;\left(-a_{p}\;|e|\right)$$

where *T*_max_, *T*_min_ and *a*_*i*_ are the positive parameters. *T*_max_ > *T*_min_ and integral time is bound by *T*_max_ ≥ *T*_*i*_ ≥ *T*_min_.

*e*_1_ is the error between infected hepatocytes and their reference value and *e*_2_ is the error between virions and their reference value, defined as:

(8) $$\left\{\begin{array}{c} e_{1}=x_{2}-x_{2_{ref}}\\ e_{2}=x_{3}-x_{3_{ref}} \end{array}\right.$$

Substituting *e*_1_ from eq. (8) in eq. (5) for control input law *u*_1_:

(9) $$u_{1}\left(t\right)\;=\;K_{p}\;e_{1}\left[e_{1}\left(t\right)\;+\;\frac{1}{T_{i}\left(e_{1}\right)}\;\int_{0}^{t}\;e_{1}\left(t\right)d\left(t\right)\;+\;T_{d}\;\frac{d\;e_{1}\left(t\right)}{dt}\right]$$

Similarly for control input *u*_2_, using eq. (8) and eq. (5):

(10) $$u_{2}\left(t\right)\;=\;K_{p}\;e_{2}\left[e_{2}\left(t\right)\;+\;\frac{1}{T_{i}\left(e_{2}\right)}\;\int_{0}^{t}\;e_{2}\left(t\right)d\left(t\right)\;+\;T_{d}\;\frac{d\;e_{2}\left(t\right)}{dt}\right]$$

### 4.3 Synergetic controller design

The synergetic theory has been proposed by Kolesnikov et al [39] where the Analytical Design of Aggregated Regulators (ADAR) method [40] is used for designing the proposed controller. Synergetic controller functions in the same way as the Sliding Mode Controller (SMC). The goal of both controllers is to force the system to operate on manifold *ψ* = 0 [41]. This control technique is capable of tracking references with an exponential rate. For this purpose, the macro‐variable is taken which contains tracking errors of the system states. By choosing the appropriate macro‐variable, interesting characteristics for the final system can be achieved according to ADAR method for synergetic such as:

- Suppression of Noise.
- Parameters insensitivity.
- Global stability.

The number of macro‐variables depends on the number of inputs of the system. In this case, number of inputs are two, so two macro‐variables were introduced as:

(11) $$\left\{\begin{array}{c} \psi_{1}=c_{1}\;e_{1}\;\\ \psi_{2}=c_{2}\;e_{2}\; \end{array}\right.$$

where *c*_1_ and *c*_2_ are real positive constants. *e*_1_ is the error between the infected hepatocytes and their reference value and *e*_2_ is the error between virions and their reference value, defined as:

(12) $$\left\{\begin{array}{c} e_{1}=x_{2}-x_{2_{ref}}\\ e_{2}=x_{3}-x_{3_{ref}} \end{array}\right.$$

Putting *e*_1_ and *e*_2_ from eq. (12) in eq. (11),

(13) $$\left\{\begin{array}{c} \psi_{1}=c_{1}\;\left(x_{2}-x_{2_{ref}}\right)\;\\ \psi_{2}=c_{2}\;\left(x_{3}-x_{3_{ref}}\right)\; \end{array}\right.$$

Taking time derivative of eq. (13),

(14) $$\left\{\begin{array}{c} {\dot{\psi }}_{1}=c_{1}\;\left({\dot{x}}_{2}-{\dot{x}}_{2_{ref}}\right)\;\\ {\dot{\psi }}_{2}=c_{2}\;\left({\dot{x}}_{3}-{\dot{x}}_{3_{ref}}\right)\; \end{array}\right.$$

Substituting ${\dot{x}}_{2}$ and ${\dot{x}}_{3}$ from eq. (3) in eq. (14),

(15) $$\left\{\begin{array}{c} {\dot{\psi }}_{1}=c_{1}\left((1-u_{1})\beta x_{3}x_{1}+r_{i}x_{2}\left(1-\frac{x_{1}+x_{2}}{T_{max}}\right)-\delta x_{2}-{\dot{x}}_{2_{ref}}\right)\;\\ {\dot{\psi }}_{2}=c_{2}\;\left((\;1\;-\;u_{2})\;p\;x_{2}-c\;x_{3}-\;{\dot{x}}_{3_{ref}}\right)\; \end{array}\right.$$

For the set of two macro‐variables, the dynamic evolution is defined as:

(16) $$\left\{\begin{array}{c} T_{1}\;{\dot{\psi }}_{1}+\;\psi_{1}=0\;\\ T_{2}\;{\dot{\psi }}_{2}+\;\psi_{2}=0\; \end{array}\right.$$

Putting ${\dot{\psi }}_{1}$ from eq. (14) in eq. (16),

(17) $$\begin{array}{ll} & T_{1}\;\left(c_{1}\;\left((1-u_{1})\;\beta \;x_{3}\;x_{1}+r_{i}\;x_{2}\left(1-\frac{x_{1}+x_{2}}{T_{max}}\right)-\delta x_{2}-{\dot{x}}_{2_{ref}}\right)\right)\;\\ & +\;\psi_{1}=0 \end{array}$$

(18) $$\begin{array}{ll} & c_{1}\;\left(\beta \;x_{3}\;x_{1}-u_{1}\;\beta \;x_{3}\;x_{1}+r_{i}\;x_{2}\left(1-\frac{x_{1}+x_{2}}{T_{max}}\right)-\delta x_{2}-{\dot{x}}_{2_{ref}}\right)\;\\ & =-\frac{\psi_{1}}{T_{1}} \end{array}$$

Solving eq. (18) for control input *u*_1_ yields:

(19) $$\begin{array}{ll} u_{1}=\left(\frac{1}{c_{1}\;\beta \;x_{3}\;x_{1}}\right) & \left(c_{1}\;\beta \;x_{3}\;x_{1}-c_{1}\;r_{i}\;x_{2}\left(1-\frac{x_{1}+x_{2}}{T_{max}}\right)\right.\\ & \left.-c_{1}\;\delta \;x_{2}-\;c_{1}\;{\dot{x}}_{2_{ref}}+\frac{\psi_{1}}{T_{1}}\right) \end{array}$$

Similarly for the macro‐variable ψ_2_, using eq. (15) and eq. (16);

(20) $$T_{2}\;\left(c_{2}\;\left((1-u_{2})\;p\;x_{2}-\;c\;x_{3}-\;{\dot{x}}_{3_{ref}}\right)\right)+c_{2}\;\left(x_{3}-x_{3_{ref}}\right)=0$$

Solving the eq. (20) for the control input law *u*_2_,

(21) $$u_{2}=\left(\frac{1}{p\;x_{2}\;c_{2}}\right)\;\left(p\;x_{2}\;c_{2}-\;c_{2}\;c\;x_{3}-\;c_{2}\;{\dot{x}}_{3_{ref}}\;+\frac{\psi_{2}}{T_{2}}\right)$$

To analyse the system's stability, the Lyapunov candidate function is taken as:

(22) $$V_{\psi_{1,2}}=\frac{1}{2}\psi_{1}^{2}\;+\;\frac{1}{2}\psi_{2}^{2}$$

Taking time derivative of $V_{\psi_{1,2}}$ in eq. (22),

(23) $${\dot{V}}_{\psi_{1,2}}={\dot{\psi }}_{1}\;\psi_{1}+{\dot{\psi }}_{2}\;\psi_{2}$$

From eq. (16), ${\dot{\psi }}_{1}$ and ${\dot{\psi }}_{2}$ can be written as:

(24) $$\left\{\begin{array}{c} {\dot{\psi }}_{1}=\frac{-\psi_{1}}{T_{1}}\\ {\dot{\psi }}_{2}=\frac{-\psi_{2}}{T_{2}} \end{array}\right.$$

Substituting ${\dot{\psi }}_{1}$ and ψ_2_ from eq. (24) in eq. (23),

(25) $${\dot{V}}_{\psi_{1,2}}=-\;\frac{\psi_{1}^{2}}{T_{1}}-\frac{\psi_{2}^{2}}{T_{2}}\leq 0$$

From eq. (25) it is clear that ${\dot{V}}_{\psi_{1,2}}$ is negative definite. So, according to Lyapunov theory, the system is stable.

As it can be seen from eq. (25), ${\dot{V}}_{\psi_{1,2}}$ is negative definite, so state variables *x*_2_ and *x*_3_ of HCV system approach to their desired reference values $x_{2_{ref}}$ and $x_{3_{ref}}$, respectively.

### 4.4 Lyapunov Redesign controller

The first step for the Lyapunov Redesign controller is to set the reference value for the infected hepatocytes and define the error as:

(26) $$\zeta_{1}=x_{2}-x_{2_{ref}}$$

where ζ_1_ is the error between infected hepatocytes and their reference value. Time derivative of eq. (26) gives:

(27) $${\dot{\zeta }}_{1}={\dot{x}}_{2}-{\dot{x}}_{2_{ref}}$$

Inserting the value of $\dot{I}$ from eq. (2) in eq. (3),

(28) $${\dot{\zeta }}_{1}=\left(1-u_{1}\right)\beta \;x_{3}\;x_{1}+r_{1}\;x_{2}\;\left(1-\frac{x_{1}+x_{2}}{T_{max}}\right)-\delta x_{2}-{\dot{x}}_{2_{ref}}$$

For stability analysis of the system and to assure the convergence of error ζ_1_ to zero, the following Lyapunov candidate function can be considered as:

(29) $$V_{1}=\frac{1}{2}\;\zeta_{1}^{2}$$

For stability, ${\dot{V}}_{1}\leq 0$. Time derivative of *V*_1_ in eq. (29) gives:

(30) $${\dot{V}}_{1}=\zeta_{1}{\dot{\zeta }}_{1}$$

By substituting values of ${\dot{\zeta }}_{1}$ from eq. (28) in eq. (30),

(31) $$\begin{array}{ll} & {\dot{V}}_{1}=\zeta_{1}\;\left((1-u_{1})\beta \;x_{3}\;x_{1}+r_{1}\;x_{2}\;\left(1-\frac{x_{1}+x_{2}}{T_{max}}\right)\right.\\ & \left.-\delta x_{2}-{\dot{x}}_{2_{ref}}\right) \end{array}$$

To make ${\dot{V}}_{1}\leq 0$ let

(32) $$\begin{array}{ll} & {\dot{\zeta }}_{1}=-k_{1}\zeta_{1}=\left(1-u_{1}\right)\beta x_{3}\;x_{1}+r_{1}x_{2}\left(1-\frac{x_{1}+x_{2}}{T_{max}}\right)\\ & -\delta x_{2}-{\dot{x}}_{2_{ref}} \end{array}$$

so that ${\dot{V}}_{1}$ becomes:

(33) $${\dot{V}}_{1}=-k_{1}\zeta_{1}^{2}$$

where *k*_1_ is the control design parameter and it should be greater than 0, so that ${\dot{V}}_{1}\leq 0$. Therefore, according to the Lyapunov's theory, the system is stable. The control input *u*_1_ for the reduction of infected hepatocytes to their reference value is derived by using eq. (32) as:

(34) $$\begin{array}{l} u_{1}=1+\frac{1}{\beta x_{3}\;x_{1}}\;\left(k_{1}\;\zeta_{1}+r_{i}\;\left(1-\frac{x_{1}+x_{2}}{T_{max}}\right)-\delta x_{2}-{\dot{x}}_{2_{ref}}\right) \end{array}$$

Similarly, an error is introduced for tracking the virions to their reference value as:

(35) $$\zeta_{2}=x_{3}-x_{3_{ref}}$$

where ζ_2_ is the error between the virions and their desired reference value. Time derivative of eq. (35) yields:

(36) $${\dot{\zeta }}_{2}={\dot{x}}_{3}-{\dot{x}}_{3_{ref}}$$

Substituting ${\dot{x}}_{3}$ from eq. (3) in eq. (36),

(37) $${\dot{\zeta }}_{2}=\left(1-u_{2}\right)\;p\;x_{2}-c\;x_{3}-{\dot{x}}_{3_{ref}}$$

For stability analysis of the system, the Lyapunov candidate function *V*_2_ is taken as:

(38) $$V_{2}=\frac{1}{2}\zeta_{2}^{2}+V_{1}$$

Taking time derivative of eq. (38),

(39) $${\dot{V}}_{2}={\dot{\zeta }}_{2}\zeta_{2}+{\dot{V}}_{1}$$

Substituting ${\dot{\zeta }}_{2}$ from eq. (37) and ${\dot{V}}_{1}$ from eq. (33) into eq. (39),

(40) $${\dot{V}}_{2}=\zeta_{2}\;\left((1-u_{2})\;p\;x_{2}-c\;x_{3}-{\dot{x}}_{3_{ref}}\right)-k_{1}\zeta_{1}^{2}$$

For ${\dot{V}}_{2}\leq 0$, let

(41) $${\dot{\zeta }}_{2}=-K_{2}\;\zeta_{2}=\left((1-u_{2})\;p\;x_{2}-c\;x_{3}-{\dot{x}}_{3_{ref}}\right)-k_{1}\zeta_{1}^{2}$$

Eq. (41) will be:

(42) $${\dot{V}}_{2}=-k_{2}\zeta_{2}^{2}-k_{1}\zeta_{1}^{2}$$

where *k*_1_ and *k*_2_ are the control design parameter and it should be a positive number. It is clear from eq. (42) that ${\dot{V}}_{2}$ is a negative definite. So, according to Lyapunov theory, the system is asymptotically stable. Solving the eq. (41) for control input law *u*_2_ is given as:

(43) $$u_{2}=1+\frac{1}{p\;x_{2}}\left(k_{2}\;\zeta_{2}-c\;x_{3}-{\dot{x}}_{3_{ref}}\right)$$

### 4.5 Fuzzy logic‐based controller (FLBC)

FLBC is the rule‐based decision‐making controller. The first step in developing a FLBC is to create a rule, based on the description of the control protocol acquired from the experts' domain instead of the accurate mathematical model.

The components of FLBC include fuzzifier, fuzzy knowledge base, fuzzy rule base, fuzzy interface system (FIS) and output defuzzification. The role of fuzzifier is to transform crisp input values into fuzzy states while fuzzy knowledge base stores the information about all the input and output fuzzy relationships. It has membership functions (MFs) which define the input variables to fuzzy rules and output variables to the plant under control. Collection of the rules is called the rule base which holds the knowledge in the form of set of rules to control the system. The rules are 'IF‐THEN' format and the 'IF' side is called 'Condition' and the 'THEN' side is called 'Conclusion'. The rule base is designed by an expert who writes a set of if‐then rules to describe what the expert thinks is the best way to control a variable. FIS is a core of FLBC which performs approximate reasoning by simulating human decisions. Defuzzification is a process of converting fuzzy values into crisp output from FIS. FLBC does not need accurate mathematical formulations.

#### 4.5.1 Fuzzy interface system

FIS is the formulation process of mapping the crisp output from a given input using the fuzzy logic. The FIS involves steps like MF, fuzzy logic operators and IF‐THEN rules. There are two types of FIS which can be implemented in the fuzzy logic toolbox: Mamdani Type‐Fuzzy Interface System (MT‐FIS) and Sugeno Type‐Fuzzy Interface System (ST‐FIS). The basic difference between MT‐FIS and ST‐FIS is based on the procedure by which the crisp output is made from the fuzzy inputs. MT‐FIS uses the defuzzification process of the output, while ST‐FIS computes the fuzzy output on the basis of the weighted average. Unlike MT‐FIS, ST‐FIS has no output MF. MT‐FIS is less flexible controller in system design which has both Multi‐ Input Single Output (MISO) and Multi‐ Input Multi‐ Output (MIMO) systems. On the other hand, the ST‐FIS is a complex system with MISO only. In this case, there are two inputs (infected hepatocytes and virions) and two outputs (*u*_1_ and *u*_2_). In this research work, MT‐FIS will be used.

#### 4.5.2 Design and implementation

FLBC has been designed by using various editors such as FIS, MF and 'IF‐THEN' rules in the Fuzzy logic toolbox. Following steps are carried out to get the desired output:

- Run MATLAB.
- Type command “FUZZY” from the MATLAB prompt to invoke Fuzzy Logic Toolbox.
- Create the fuzzy logic designer in FIS Editor: name it (In this study, it was named as HCV_controller), choose MT‐FIS as FIS (input variable are error and integral error of infected hepatocytes and virions and output variable are drug dose *u*_1_, *u*_2_).
- Define the MFs for each input and output variables in the MF Editor.
- Interpret the Fuzzy System Rules by using the Rule Editor.
- Save the model to a file (HCV_controller.fis).
- Verify the model in the Rules Viewer.
- Export and save the current FIS Model to the workspace.

The FIS Editor describes details about input variables (infected hepatocytes and virions), output variables (*u*_1_ and *u*_2_), MF's, model type and rules. The number and types of the MFs for each input and output variables such as Gaussian, Trapezoidal and Triangle are chosen in the Fuzzy Membership Function Editor. Researchers use triangular shape MF for the computation to make it relatively simple and accurate.

**FIGURE 4:**
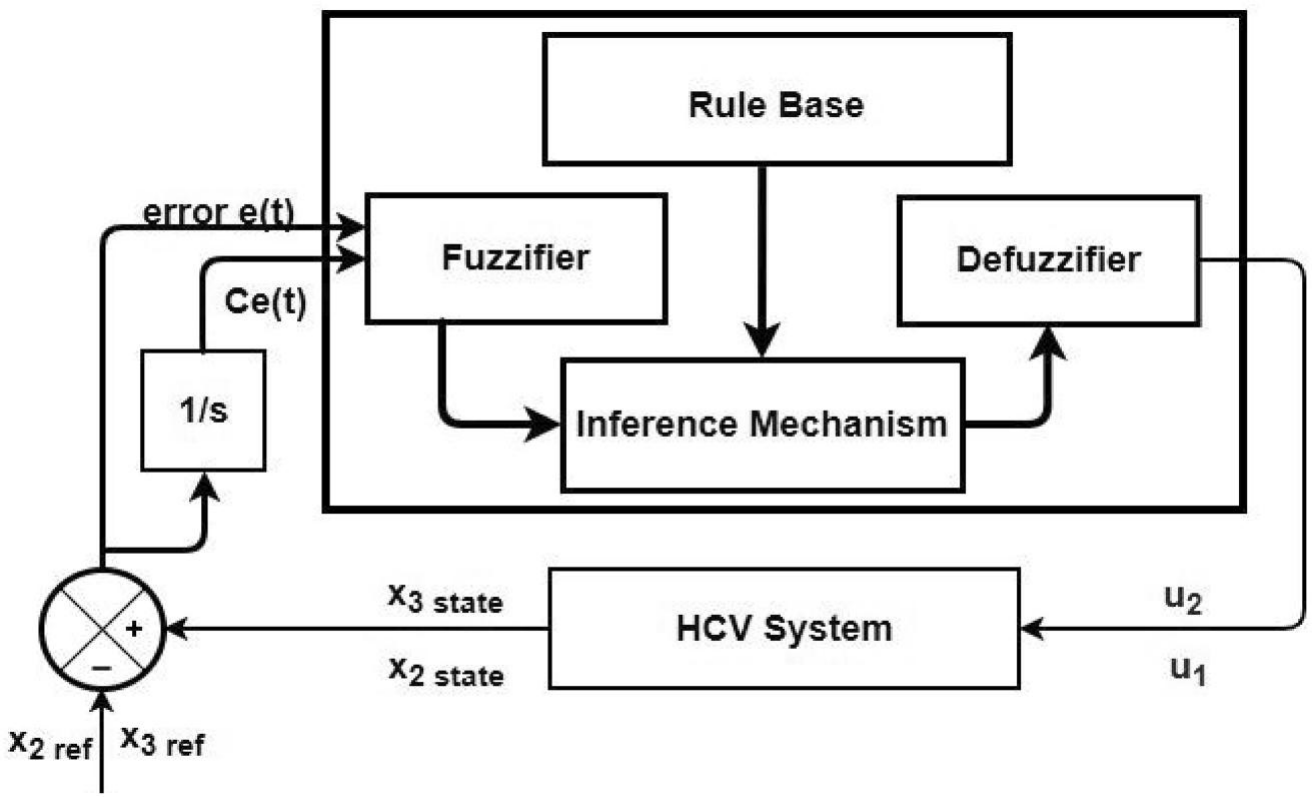
FLBC block diagram for HCV system

There are two inputs for each tracking state of the infected hepatocytes and virions. Based on these inputs, the outputs are sent. The first input is the error between the infected hepatocytes and virion state with their reference values. The second input is the change of error *Ce*(*t*) given by:

(44) $$\left\{\begin{array}{c} e\left(t\right)=\;x_{2}\;-\;x_{2_{ref}}\;\\ Ce\left(t\right)=\;e\left(t\right)\;-\;e\left(t-1\right)\; \end{array}\right.$$

and

(45) $$\left\{\begin{array}{c} e\left(t\right)=\;x_{3}\;-\;x_{3_{ref}}\;\\ Ce\left(t\right)=\;e\left(t\right)\;-\;e\left(t-1\right)\; \end{array}\right.$$

where 't' is the sampling time. *e*(*t*) is the error resulting from the subtraction of the infected hepatocytes and virions from their reference values which are sampled with time t. *C e*(*t*) is the change of error produces from subtraction of current error and previous error. The outputs of the fuzzy controller in the case of infected hepatocytes are the anti‐viral drug dose peg‐IFN‐*α* as *u*_1_ and ribavirin as *u*_2_ which are fed to the HCV system as control signals shown in Figure 4.

**FIGURE 5:**
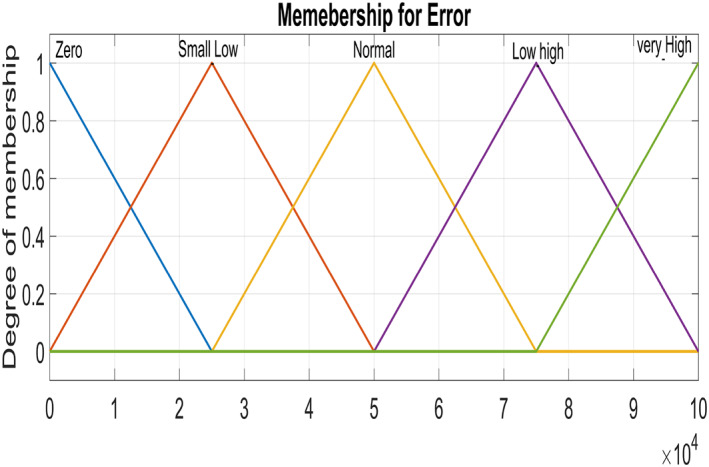
Input variable ‐ Error for Virions

**FIGURE 6:**
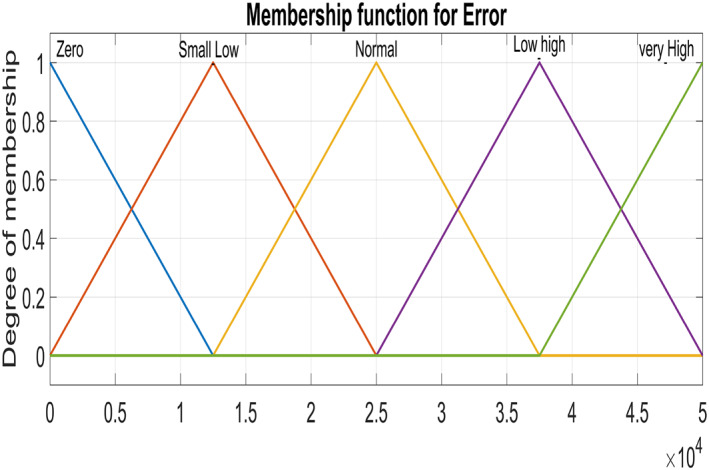
Input variable ‐ Error for Infected Hepatocytes

**FIGURE 7:**
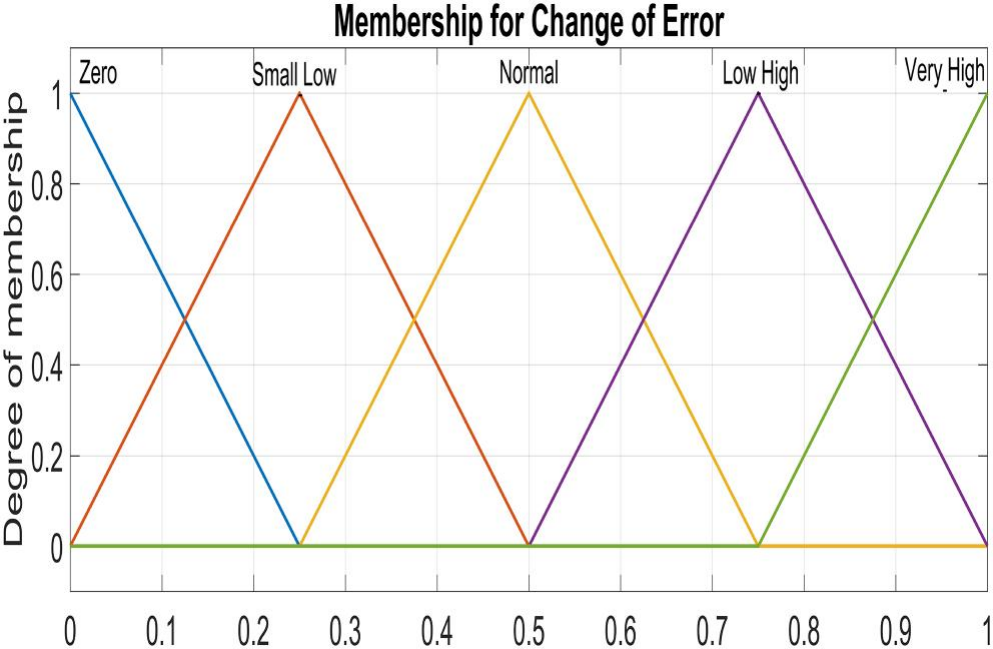
Input Variable ‐ Change of error

**FIGURE 8:**
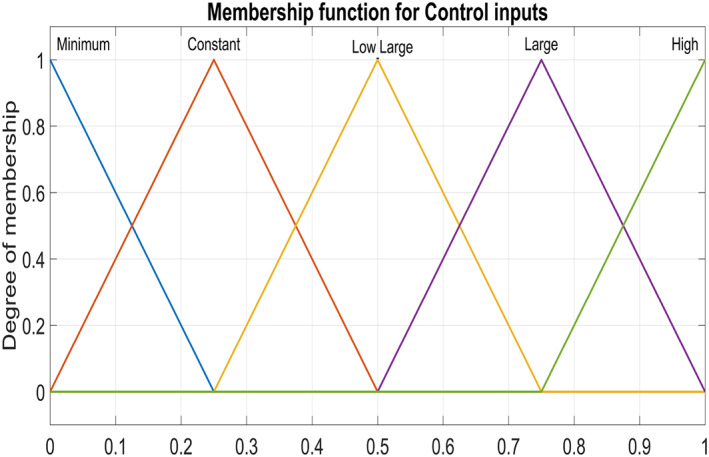
Output Variable ‐ control inputs for both states

Figure 5 and Figure 6 show triangular MFs for input variables *e*(*t*) while Figure 7 show striangular MFs for input variables *Ce*(*t*) for both states. Each input variables has five triangular MFs. The output variables *u*_1_ for the infected hepatocytes and *u*_2_ for the virions also have five triangular MFs each as shown in Figure 8.

The Fuzzy system rules have been designed in Rule Editor. The derivation of fuzzy rules are of heuristic nature and based on the following criteria:

- IF *e*(*t*) and *Ce*(*t*) for infected hepatocytes and virions are far away from the reference value, 0 drug dose should be large enough to bring them to their desired reference value.
- IF *e*(*t*) and *Ce*(*t*) for infected hepatocytes and virions are converging towards the reference value, THEN a small change in the drug dose is obligatory.
- IF the desired reference value is achieved and steady, THEN the drug dose should keep constant.
- IF *e*(*t*) and *Ce*(*t*) for infected hepatocytes and virions are greater than those of the reference value, THEN the drug dose should be minimum to achieve the reference value.

Fuzzy rules for drug dose are mentioned in Table 2. The simulation of the Fuzzy rules for FLBC is done by using MATLAB/Simulink toolbox. MT‐FIS FLBC is used for the implementation of this work.

**TABLE 2:** FLBC rule formation table

Change of Error	Error				
	Zero	Small low	Normal	Low high	Very high
Zero	Minimum	Minimum	Minimum	Constant	Low large
Small low	Minimum	Minimum	Constant	Low large	Large
Normal	Minimum	Constant	Low large	Large	High
Low high	Low large	Low large	Large	High	High
Very high	Constant	Large	High	High	High

## 5 SIMULATION RESULTS

The MATLAB/Simulink‐based environment has been used for showing the performance of the proposed control input laws given by the eqs. (9) and (10) for non‐linear PID, eqs. (19) and (21) for the Synergetic controller, by eqs. (34) and (43) for the Lyapunov Redesign controller and by the fuzzy rules for MT‐FIS for treatment period of 90 days. The initial conditions at the start of the chronic stage of HCV for uninfected hepatocytes, infected hepatocytes and virions were *x* = [555 *cells*/*mL*, 50, 000 *cells*/*mL*, 100, 000 *cells*/*mL*]^*T*^ , respectively. These values were calculated by a test called polymerase chain reaction (PCR) which is used to measure the amount of virions, infected hepatocytes and uninfected hepatocytes in the blood of the HCV patient. Then the control was applied to regulate the uninfected hepatocytes, infected hepatocytes and the virions' state in order to reach the accepted condition at the equilibrium point of *x* = [8000000,0,0]^*T*^ at the end of treatment period for 90 days. The desired reference values for the uninfected hepatocytes, infected hepatocytes and virions were set at *T*_max_ = 8,000,000, $x_{2_{ref}}$ = 0, $x_{3_{ref}}$ = 0, respectively. All the parametric values used in the HCV dynamical model are taken from [42] and are listed in Table 3. The design parameters for the proposed controllers have been set up by the trial and error method. The gain value can be changed until the required reference is achieved. Linear PID controller design parameters have been obtained using the auto‐tune method available in MATLAB/Simulink. The gain parametric values of Synergetic, Lyapunov Redesign, non‐linear PID and linear PID controllers have been shown in Table 4.

**TABLE 3:** Parameters and values of variables for non‐linear HCV System

Parameter	Values
*s*	0.0024 * 10^7^
*d*_*T*_	0.003
*β*	10^−7^
*c*	10
*δ*	0.2
*p*	20
*r*_*T*_	2.0
*r*_*i*_	1.0

**TABLE 4:** Gain parameters values

Controller	Varaible	Value
**Linear PID**	*K*_*p*_	0.1
	*K*_*i*_	0
	*K*_*d*_	1
**Non‐linear PID**	*K*_max_	10
	*K*_min_	5
	*a*_*p*_	250
	*T*_max_	0.0115
	*T*_min_	0.0086
	*a*_*i*_	250
**Synergetic controller**	*C*_1_	780
	*T*_2_	1
	*T*_1_	0.000005
	*C*_2_	0.11
**Lyapunov Redesign controller**	*k*_1_	0.5
	*k*_2_	1

**FIGURE 9:**
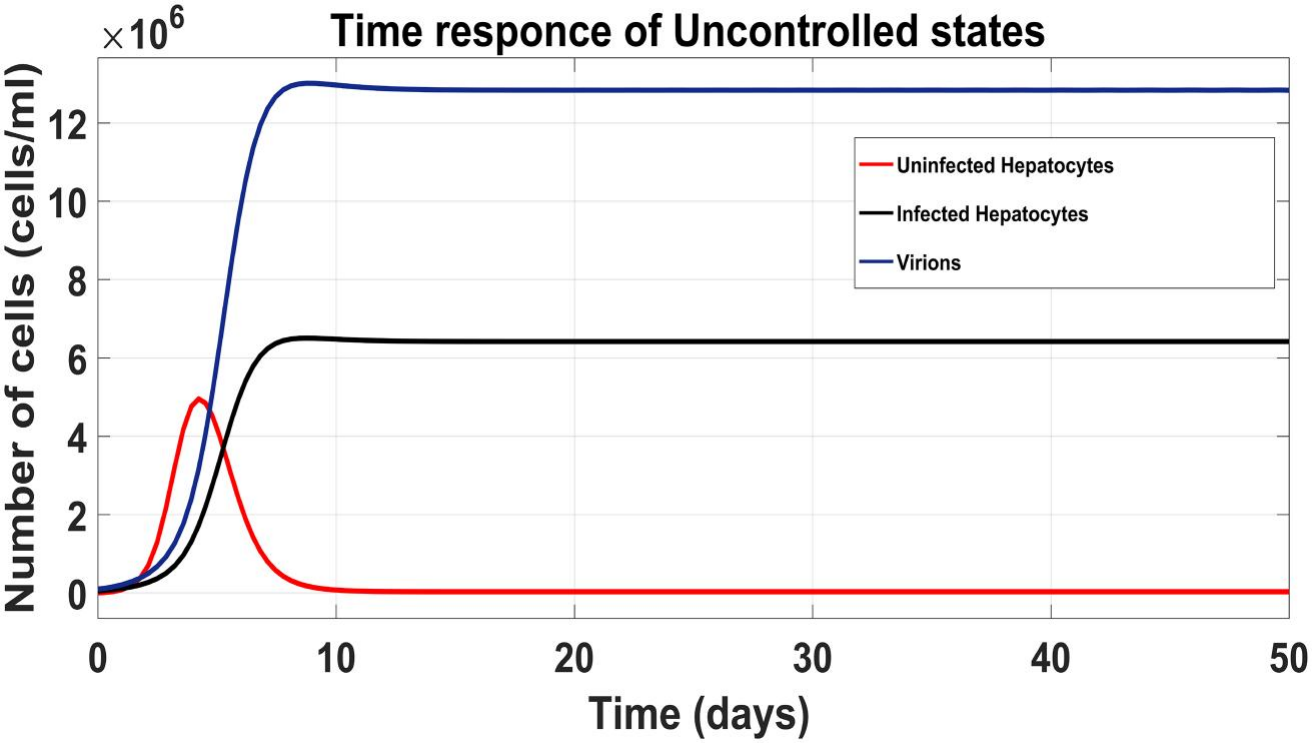
Uncontrolled response of all states of HCV

Figure 9 shows the trend of virions, infected hepatocytes and uninfected hepatocytes (uncontrolled response) in which the virions and the infected hepatocytes have a substantial increase in the early period of the disease. Due to the high concentration of infected hepatocytes and virions, uninfected hepatocytes are reduced. Although, using the proposed Synergetic controller, Lyapunov Redesign controller, FLBC and non‐linear PID for drug injection by control input laws, the concentration of the uninfected hepatocytes increase to their maximum limit *T*_max_, while infected hepatocytes and virions reduce and decrease to their reference values.

According to control inputs, it is obvious that the drug efficacy *u*_1_ must be at the highest level for a shorter period of time during the first phase of the disease after exposure to the virus. Then drug efficacy decreases after certain level for second 'shoulder phase' and remains at the minimum level for the rest of treatment period. The maximum value for the drug efficacy *u*_1_ is set at 0.96, so the total amount of that drug can be used within the safe limits. However, drug efficacy *u*_2_ supposed to be lower at first phase of treatment and after that control increases rapidly again to the maximum safe limit of 0.96 and remains at this level for the rest of treatment period.

This section consists of a further four subsections. Subsection‐A shows the comparison performance of Synergetic controller with linear and non‐linear PID controllers. The performance of the Synergetic controller has been compared with that of the Lyapunov Redesign controller in the subsection‐B while the subsection‐C contains the comparison of the Synergetic controller with the FLBC. In subsection‐D, all the proposed controllers are compared with each other on the basis of the transient response, settling time, overshoots/undershoots, SSE and magnitude of ripples in their performance. Performance of proposed controllers subjected to measurement noise is discussed in subsection‐E.

### 5.1 Comparison of synergetic controller with nonlinear PID and linear PID controller

**FIGURE 10:**
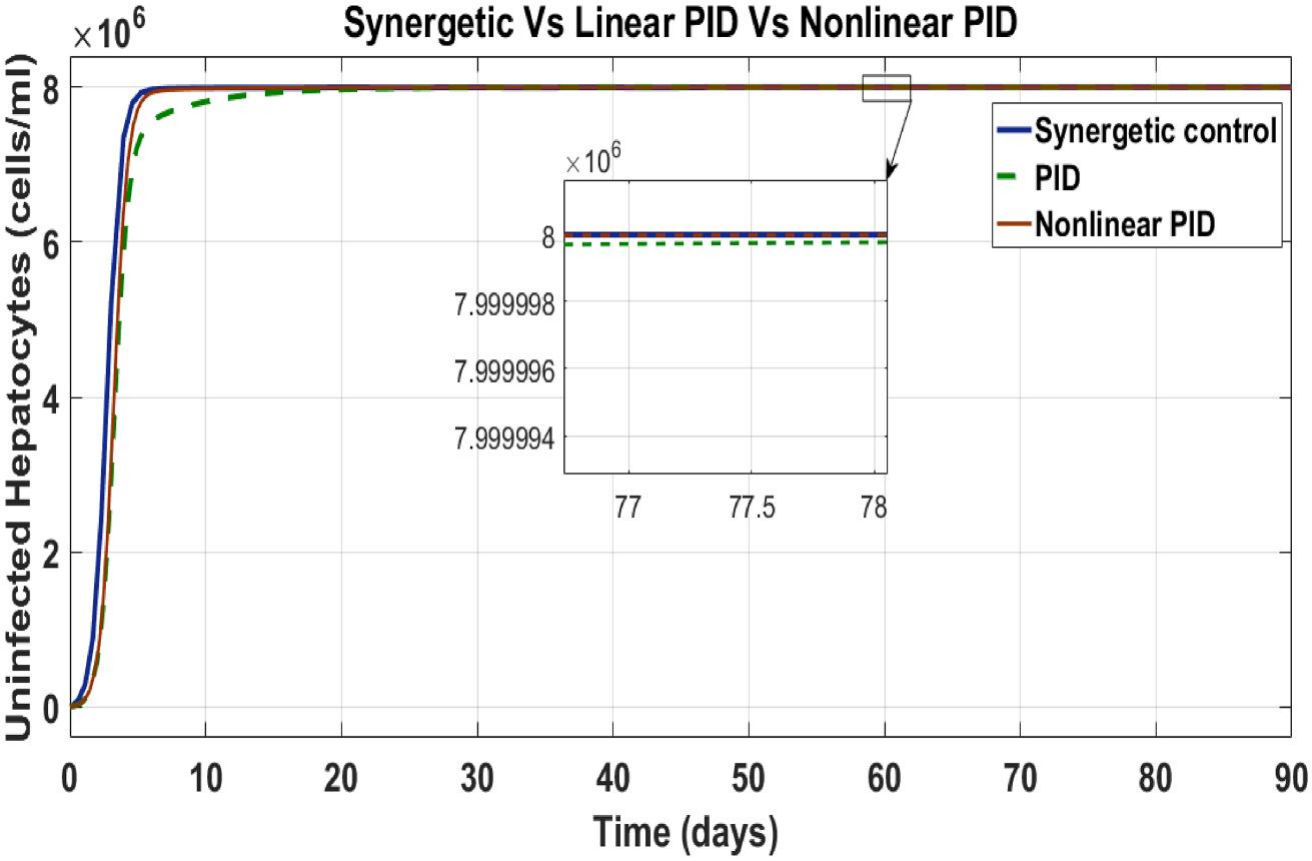
Behaviour of uninfected hepatocytes

Here, the comparison of the Synergetic with non‐linear and linear PID controller for the uninfected hepatocytes, infected hepatocytes and virions of the HCV has been made. Behaviour of the uninfected hepatocytes for the Synergetic and the PID controller is shown in Figure 10. The uninfected hepatocytes grow faster, show remarkable increase and achieve *T*_max_, the maximum concentration of the uninfected hepatocytes inside the liver. The transient time of the uninfected hepatocytes to *T*_max_ is better with the Synergetic controller as compared with that of the PID controllers. SSE has been observed in the uninfected hepatocytes with the linear PID, while the Synergetic controller and non‐linear PID controller achieves *T*_max_ limit with no SSE.

**FIGURE 11:**
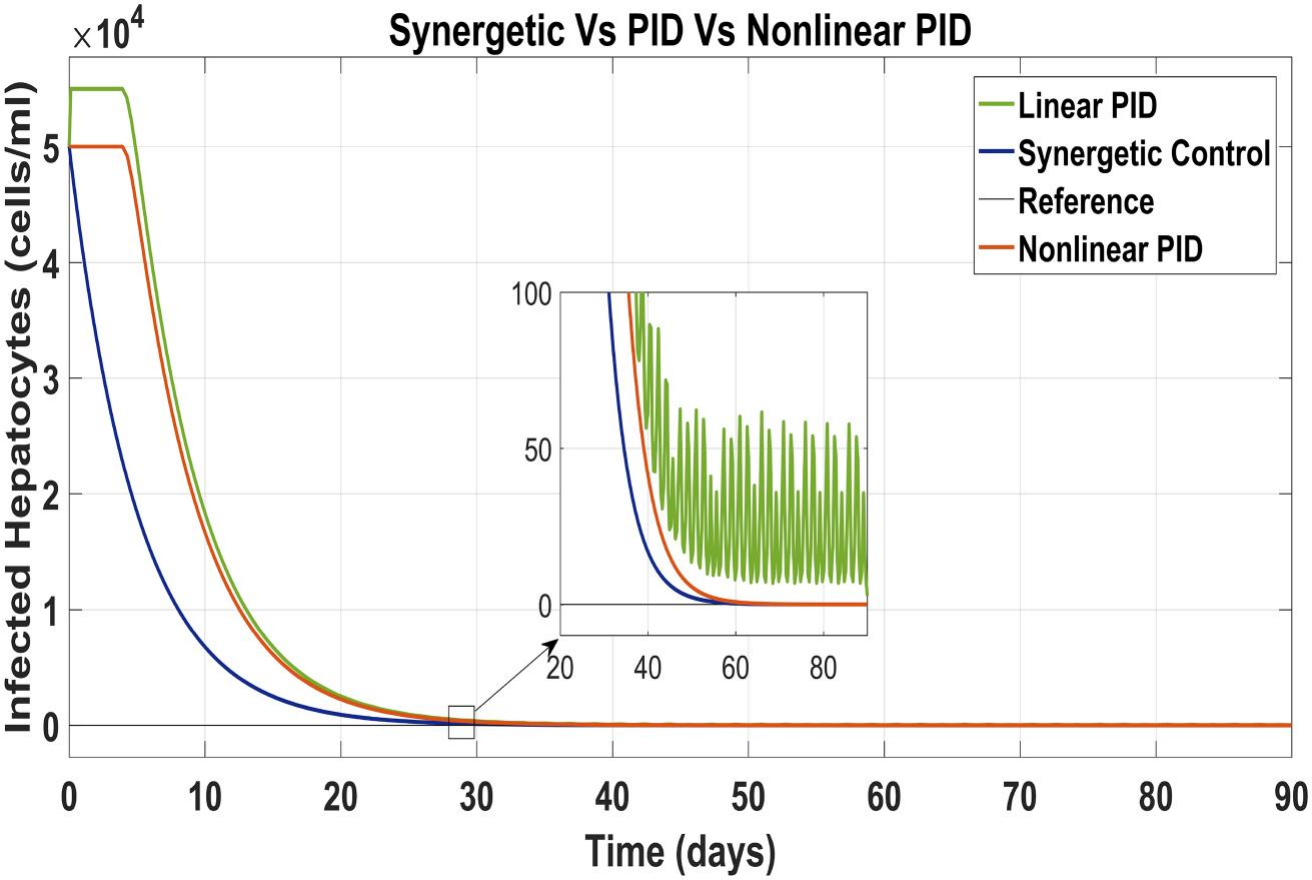
Behaviour of infected hepatocytes

Figure 11 has been drawn to show the behaviour of the infected hepatocytes for the proposed controllers which reveals the fast tracking time of the infected hepatocytes by the control input *u*_1_ to its reference value with the Synergetic controller as compared with that of the PID controller. SSE and overshoots have been observed in tracking the reference value by using linear PID. The infected hepatocytes track the reference value in 55 and 61 days having no SSE with the Synergetic controller and the non‐linear PID, respectively.

**FIGURE 12:**
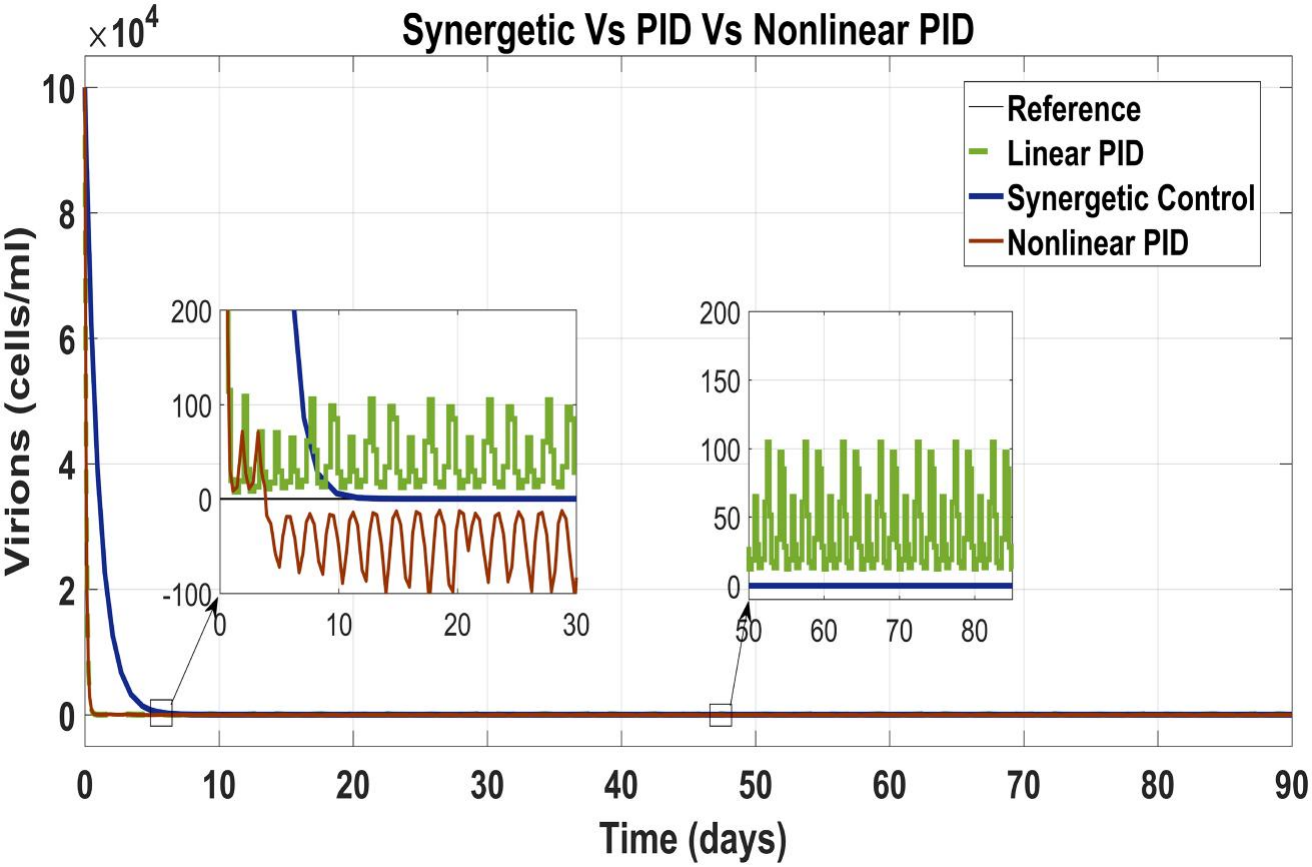
Behaviour of the virions

Figure 12 shows behaviour of virions which track the reference value by control input *u*_2_ as anti‐viral drug. They track their reference values very quick with the Synergetic controller as compared with that of the PID controllers. The tracking time of the virions is 30 days with the Synergetic controller with no SSE using anti‐viral drugs, while linear and non‐linear PID controllers shows SSE of about 10 cells in the tracking virions.

**FIGURE 13:**
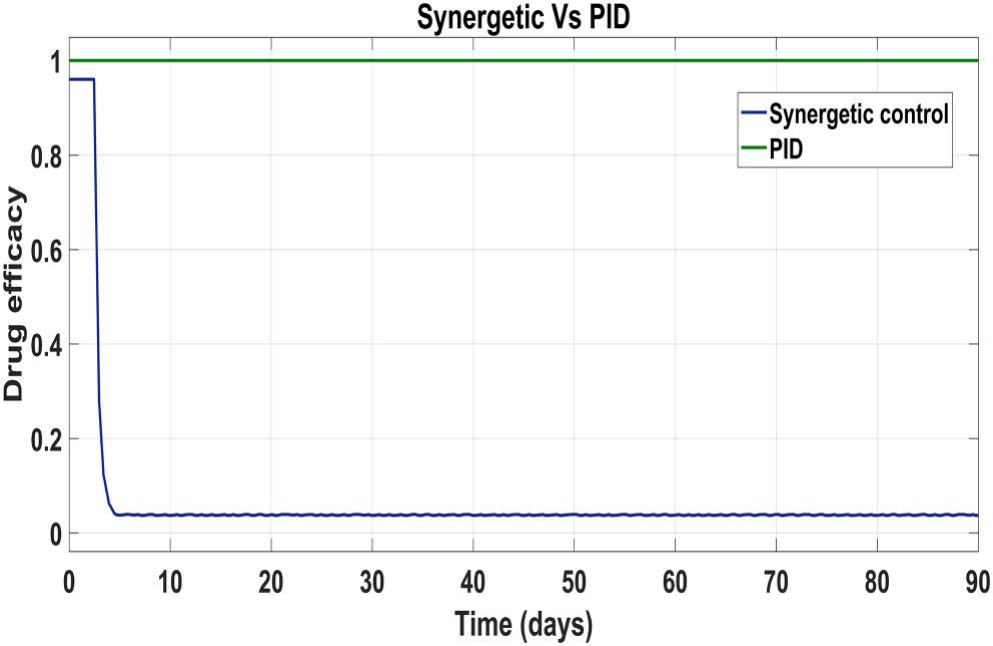
Control input *u*_1_ comparison

**FIGURE 14:**
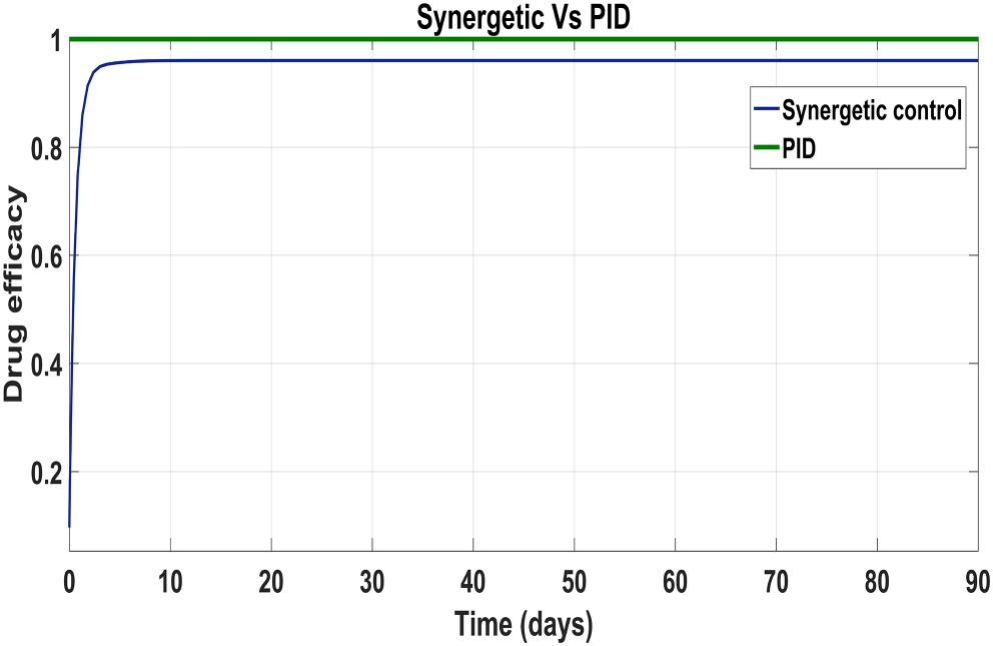
control input *u*_2_ comparison

Behaviour of the drug efficacy for both the control inputs is shown in Figure 13 and Figure 14 using both controllers. This comparison shows that drug injection by both the linear and the non‐linear PID controllers remains high throughout the treatment period. It is also observed that the drug injection *u*_1_ remains low at the start of therapy within the safe limit with the Synergetic controller as compared with the PID controller. Drug dose *u*_2_ remains high at the start of therapy and reaches to efficient value for the rest of treatment period.

Figures 11 and 12 clearly show that under the proposed treatment strategy, infected hepatocytes and virions track their desired reference values with the Synergetic controller. From these figures, it is inferred that the proposed treatment strategy is effective; hence, asymptotic stability has been achieved.

### 5.2 Comparison of synergetic controller with Lyapunov Redesign controller

**FIGURE 15:**
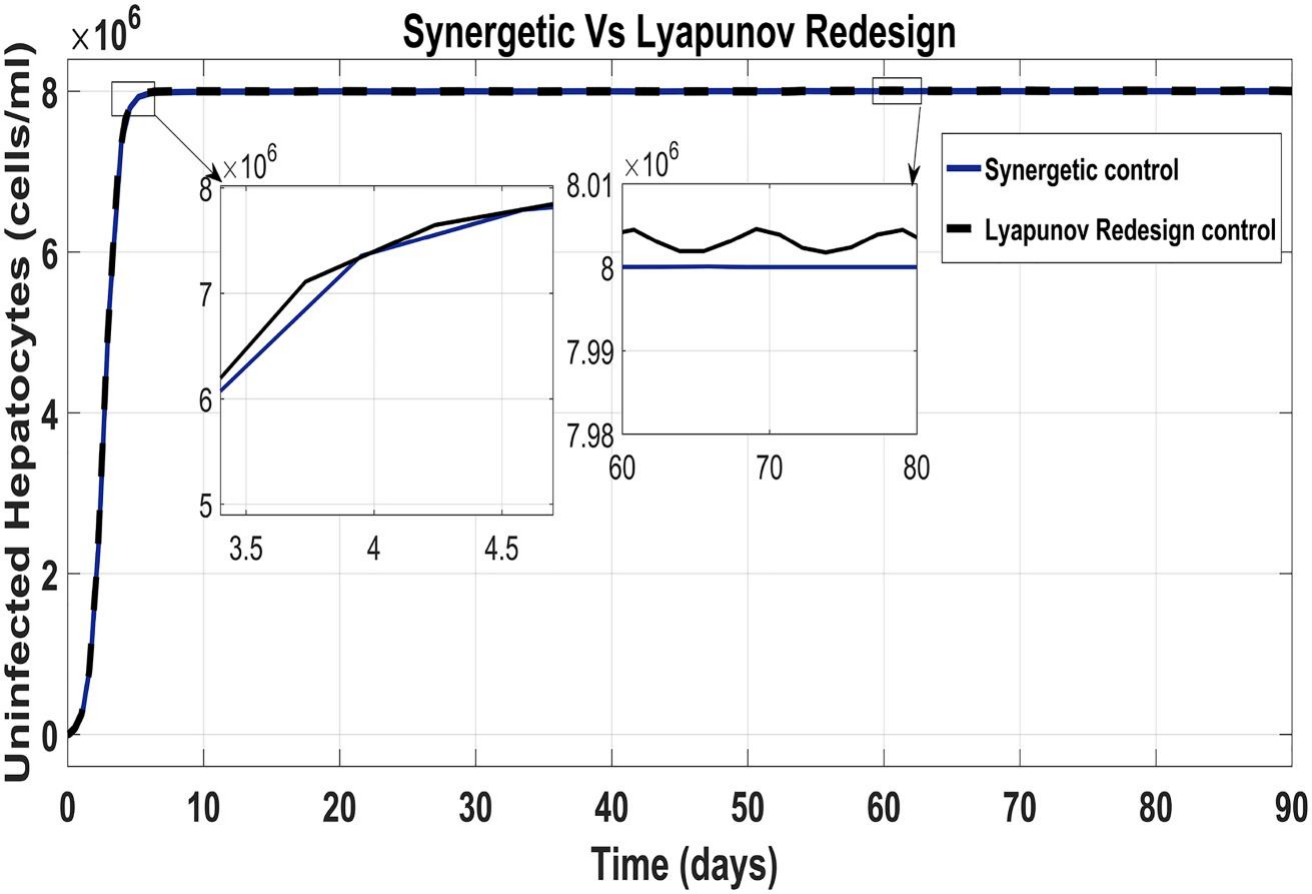
Behaviour of the uninfected hepatocytes

The behaviour of the uninfected hepatocytes using the Synergetic controller and the Lyapunov Redesign controller has been shown in Figure 15. It has been observed that the uninfected hepatocytes approach *T*_max_ quickly using the Lyapunov Redesign controller as compared with that of the Synergetic controller. However, the Lyapunov Redesign controller observes huge ripples and overshoots in achieving *T*_max_. This results in the enlargement of liver called hepatomegaly. This creates serious health problems like cancer such as leukemia, heart abnormalities and genetic diseases, etc. So maintaining uninfected hepatocytes at *T*_max_ is necessary. The approach time of the uninfected hepatocytes to *T*_max_ shows no SSE and ripples with the Synergetic controller.

**FIGURE 16:**
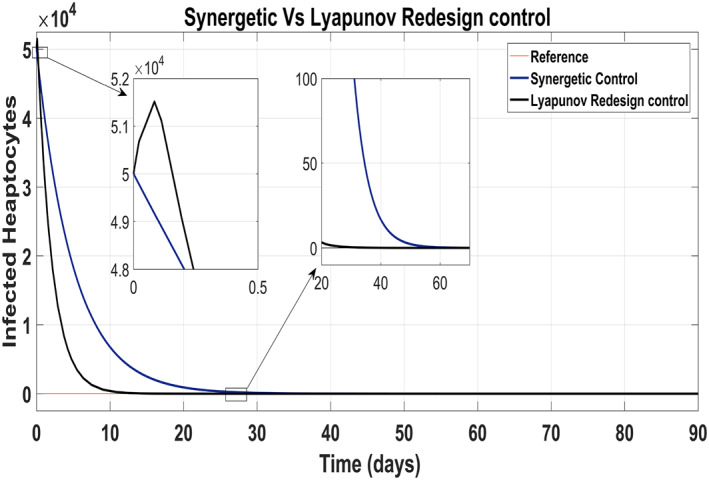
Behaviour of infected hepatocytes

Tracking of infected hepatocytes to their reference value is shown in Figure 16 where it is clearly shown that the tracking time of the infected hepatocytes to the reference value is better with the Lyapunov Redesign controller than that with the Synergetic controller.

Infected hepatocytes approach to zero reference in 30 days using antiviral drugs with the Lyapunov Redesign controller, whereas 55 days with the Synergetic controller by maintaining their reference value for the rest of the treatment period. Initially, the Lyapunov Redesign controller observes some overshoots of the anti‐viral therapy.

**FIGURE 17:**
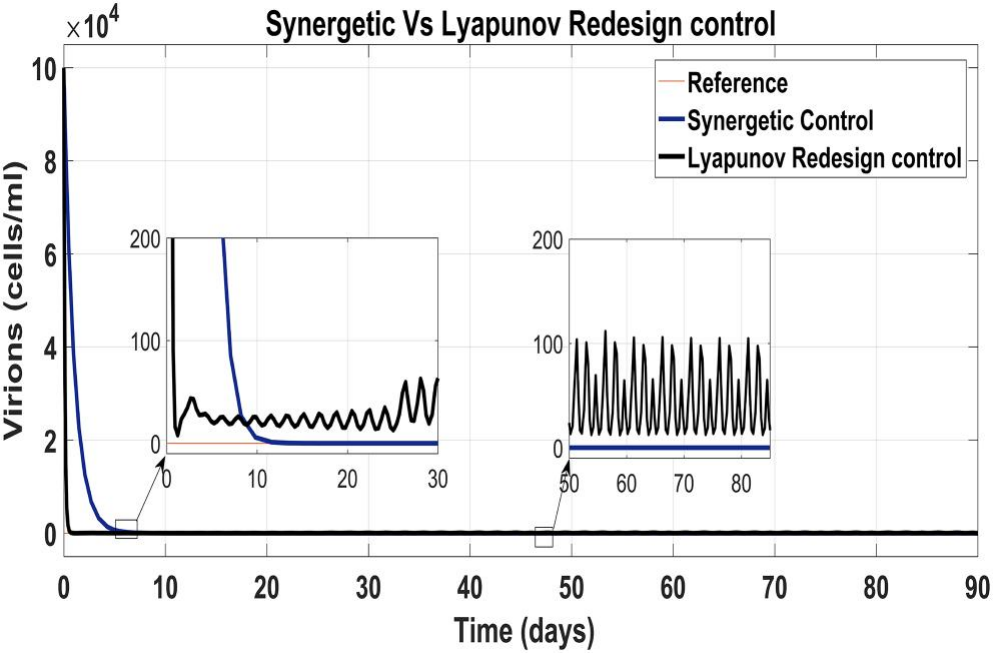
Behaviour of virions

**FIGURE 18:**
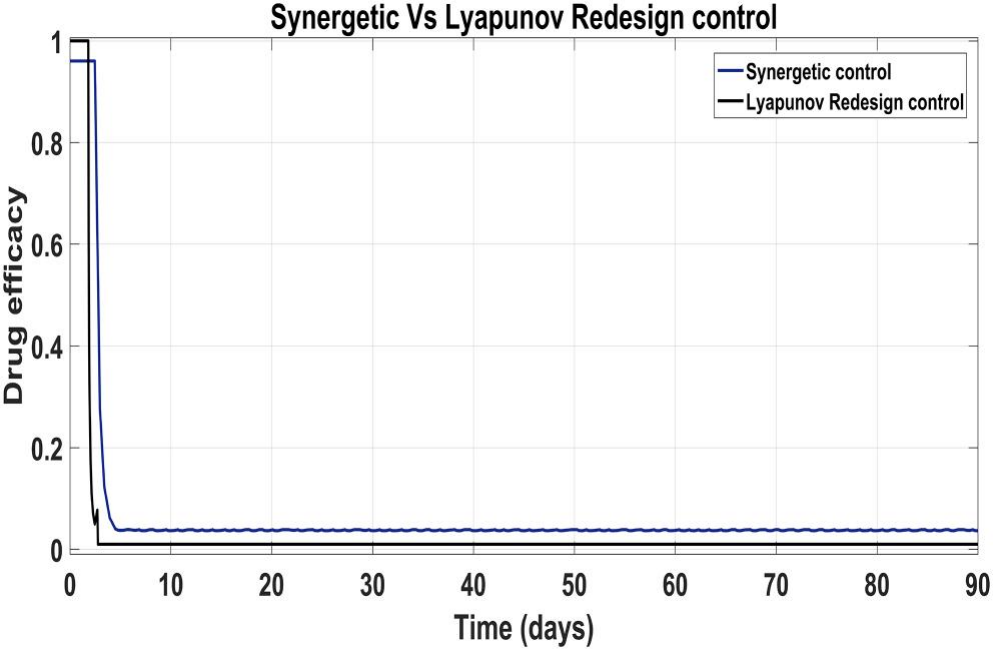
Control input *u*_1_ comparison

**FIGURE 19:**
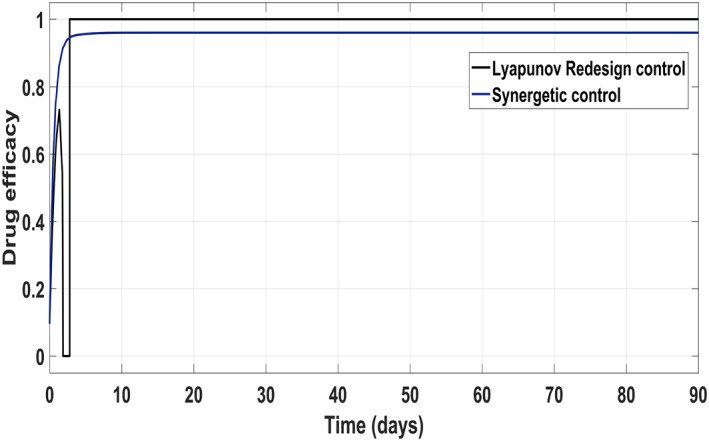
Control input *u*_2_ comparison

Figure 17 shows the suppression of virions to their reference value. Again, the results are more efficient using the Synergetic controller carrying no SSE and ripples while the Lyapunov Redesign controller shows substantial SSE and ripples. Both controllers have slight difference in injection of drugs (shown in Figures 18 and 19). Simulation results show that lesser amount of drug is used in therapy with the Synergetic controller as compared to the Lyapunov Redesign controller.

### 5.3 Comparison of the Synergetic controller with the FLBC

**FIGURE 20:**
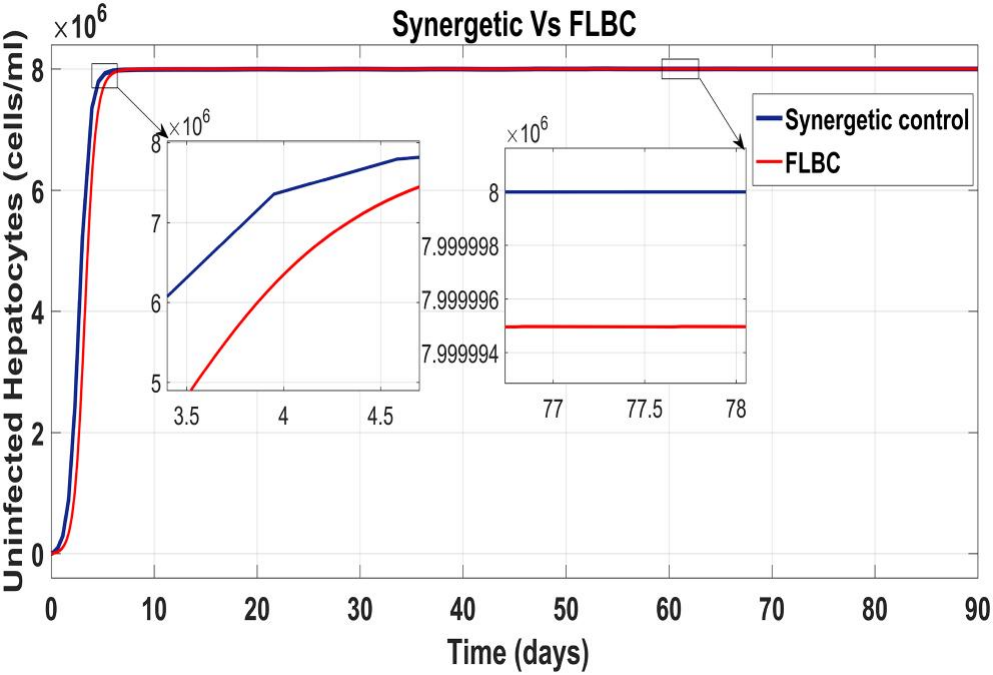
Behaviour of uninfected hepatocytes

**FIGURE 21:**
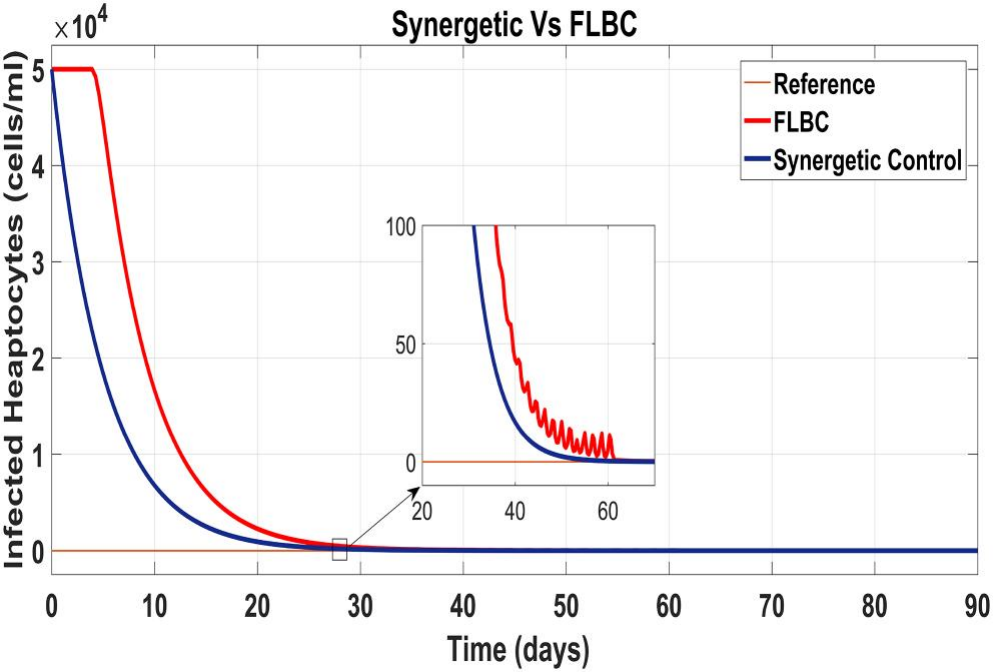
Behaviour of the infected hepatocytes

Figure 20 shows the concentration of the uninfected hepatocytes after usage of the drug for the proposed controllers. The transient time of the uninfected hepatocytes to the maximum limit *T*_max_ is better for the Synergetic controller. The FLBC shows SSE in achieving *T*_max_. Figure 21 shows that the infected hepatocytes track their reference value more quickly with no SSE, oscillations and overshoots/undershoots using the Synergetic controller. The FLBC shows slow tracking of the infected hepatocytes with the oscillations.

**FIGURE 22:**
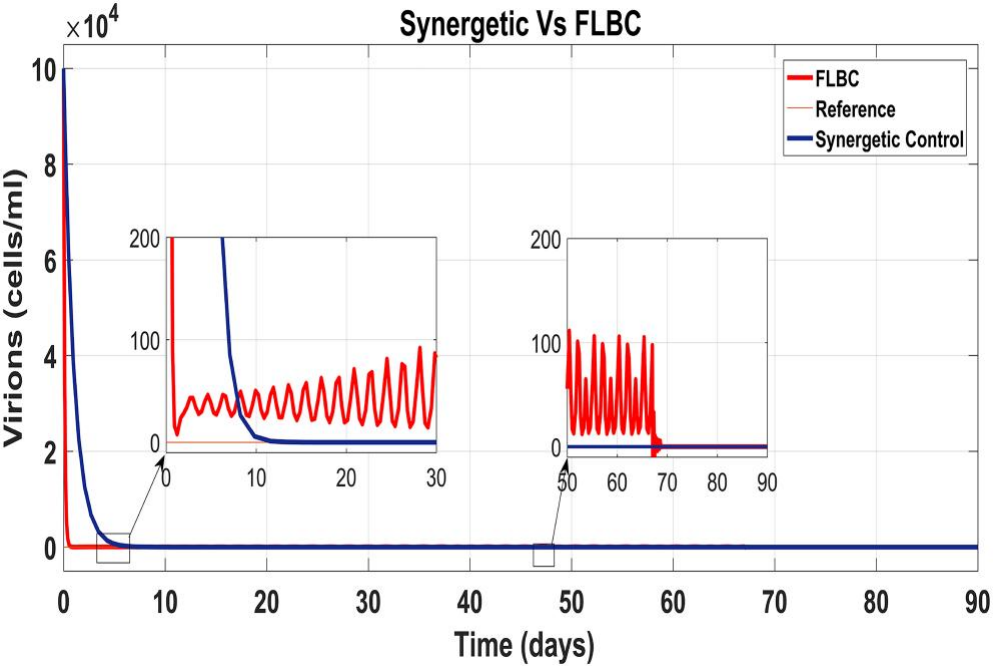
Behaviour of the virions

**FIGURE 23:**
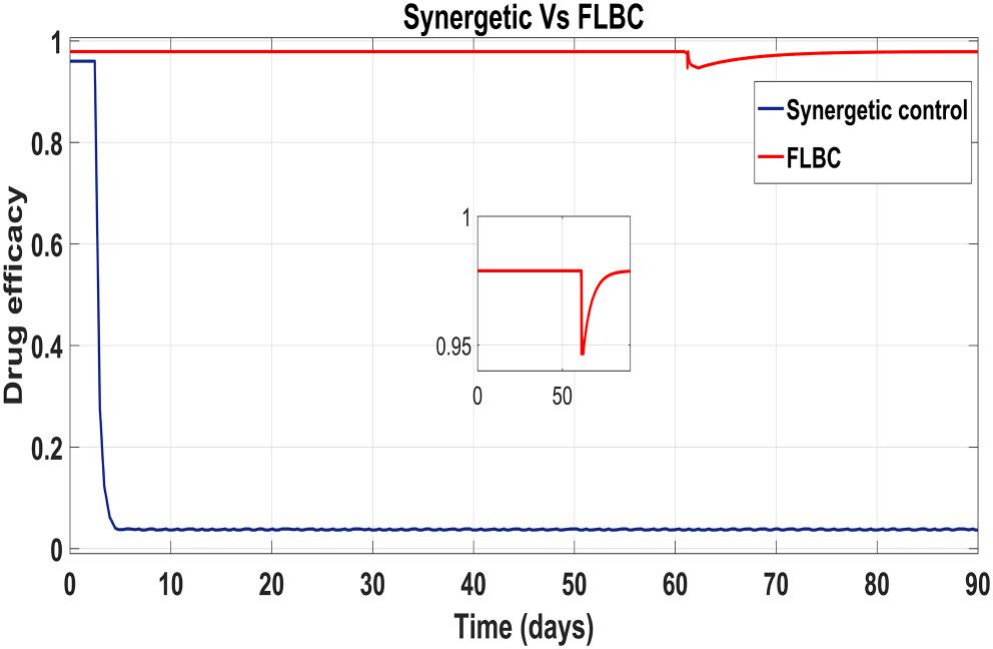
Comparison of the control input *u*_1_

**FIGURE 24:**
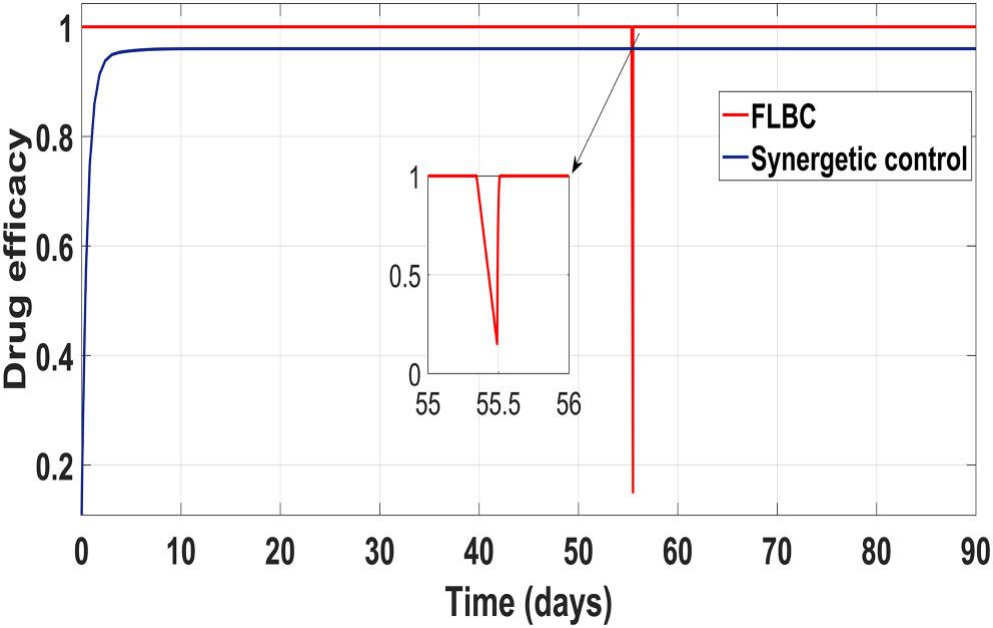
Comparison of the control input *u*_2_

Figure 22 shows that the approach time of the virions to their reference value is faster with the Synergetic Controller than with FLBC. Oscillations have been observed in tracking virions to their reference value. Synergetic controller eliminates SSE, overshoots/undershoots and oscillations completely. Drug dose remains high throughout anti‐viral therapy with both control inputs *u*_1_ and *u*_2_ using FLBC (Figure 23 and 24). It is cleared that all the control objectives are satisfied using the Synergetic controller.

### 5.4 Comparison between proposed controllers

In this subsection, comparison of the performance of all the proposed controllers with each other and with the linear PID on the basis of undershoots/overshoots, SSE, transient response, settling time and oscillations has been carried out, while tracking their respective reference values. It has been observed that transient time of uninfected hepatocytes to *T*_max_ is 52 days and settling time is about 58 days with no SEE, ripples, oscillations and overshoots. The virions and infected hepatocytes approach to their reference value within 55 and 30 days, respectively, with no SSE, ripples, overshoots/undershoots and oscillations using the Synergetic controller.

Ripples, oscillations and SSE have been observed in the uninfected hepatocyte and virion state of the HCV using the Lyapunov Redesign controller. The transient time of the uninfected hepatocytes to *T*_max_ is 62 days while settling time is 70 days. The Lyapunov Redesign provides better convergence time for the infected hepatocytes to their reference value than the other proposed controllers. The infected hepatocytes converge to their reference in 30 days by injecting the anti‐viral drugs through the control input.

The FLBC shows oscillations and huge undershoots/overshoots in the infected hepatocytes and virions in all states of the HCV system while tracking their respective references. The transient and settling time for uninfected hepatocytes to the maximum limit is 40 and 70 days, respectively. It takes 62 to 67 days to track the infected hepatocytes and virions to their reference values. The PID shows a poor transient and settling time as compared with the proposed non‐linear controllers.

The non‐linear PID shows no SEE, overshoots/undershoots and oscillations in the uninfected and infected hepatocyte states. Virions shows SSEs of about 20 cells, 100 cells in oscillation and 30 cells undershoot/overshoot. The transient time of the uninfected hepatocytes to *T*_max_ is 60 days while settling time is 61 days. It takes 65 to 74 days to track the infected hepatocytes and virions to their reference values.

The values of the SSE, oscillations, undershoots/overshoots, transient time and settling time for all the proposed controllers and the PID are given in Table 5 for uninfected hepatocytes, infected hepatocytes and virions state.

**TABLE 5:** Response of the proposed controllers

Uninfected hepatocytes					
Response	PID	Non‐linear PID	Synergetic controller	Lyapunov Redesign controller	FLBC
SSE (cells)	9000	0	0	50,000	20,000
Overshoot/Undershoot	0	0	0	100,000	0
Oscillations (cells)	0	0	0	0	0
Settling time (day)	65	61	58	70 or more	70 or less
Transient time (day)	62	60	52	62	40

**Infected hepatocytes**					
Response	PID	Non‐linear PID	Synergetic controller	Lyapunov Redesign controller	FLBC
SSE (cells)	15	0	0	0	0
Overshoot/Undershoot	50,000	0	0	1500	0
Oscillations (cells)	10	0	0	0	15
Settling time (days)	70	65	52	28	62
Transient time (days)	65	61	50	27	60

**Virions**					
Response	PID	Non‐linear PID	Synergetic controller	Lyapunov Redesign controller	FLBC
SSE (cells)	20	20	0	10	0
Overshoot/Undershoot	20	20	0	30	0
Oscillations (cells)	100	100	0	120	50
Settling time (days)	70 or more	72	27	60 or more	67
Transient time (days)	70 or more	74	18	60 or more	65

### 5.5 Performance of the proposed controllers under measurement noise

In order to evaluate the efficiency of proposed treatment under the measurement noise *v*_*k*_, the white noise with a magnitude of 10% as shown in (Figure 26) has been added to the output values of the measurement. The model has been given by:

(46) $$\left\{\begin{array}{cc} \frac{dx_{1}}{dt} & =\;s+r_{T}\;x_{1}\left(1-\frac{x_{1}+x_{2}}{T_{max}}\right)-d_{T}x_{1}-\left(1-u_{1}\right)\beta \;x_{3}x_{1}+v_{k}\\ \frac{dx_{2}}{dt} & =\;\left(1-u_{1}\right)\beta \;x_{3}\;x_{1}+r_{i}\;x_{2}\;\left(1-\frac{x_{1}\;+\;x_{2}}{T_{max}}\right)-\delta \;x_{2}+v_{k}\\ \frac{dx_{3}}{dt} & =\;\left(1-u_{2}\right)\;p\;x_{2}-\;c\;x_{3}+v_{k} \end{array}\right.$$

**FIGURE 25:**
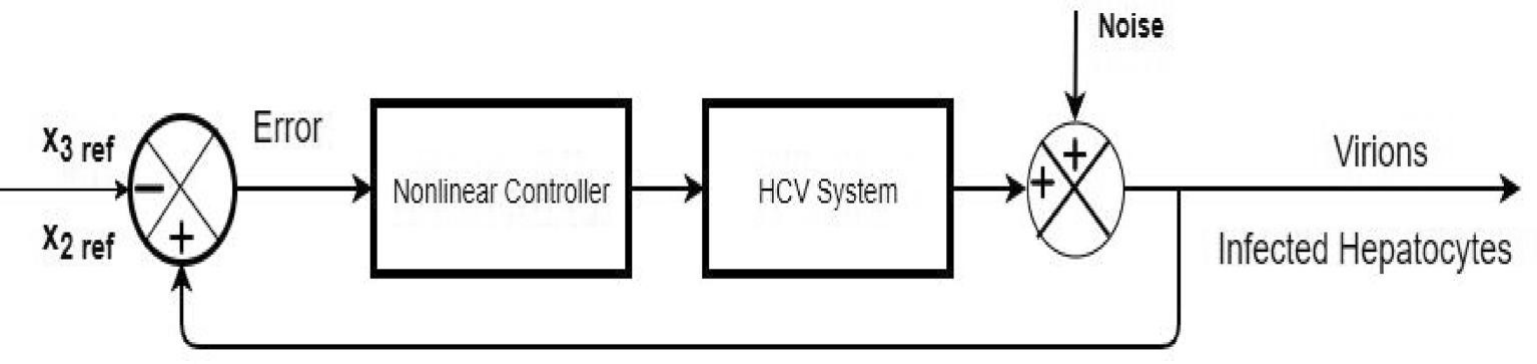
Closed loop block diagram for HCV in case of noise

Figure 25 shows the control block diagram of the HCV system in the presence of noise. The comparative analysis of all the proposed non‐linear controllers and PID in the presence of noise have been shown in Figure 27 for uninfected hepatocytes, Figure 28 for the infected hepatocytes and Figure 29 for the virions.

**FIGURE 26:**
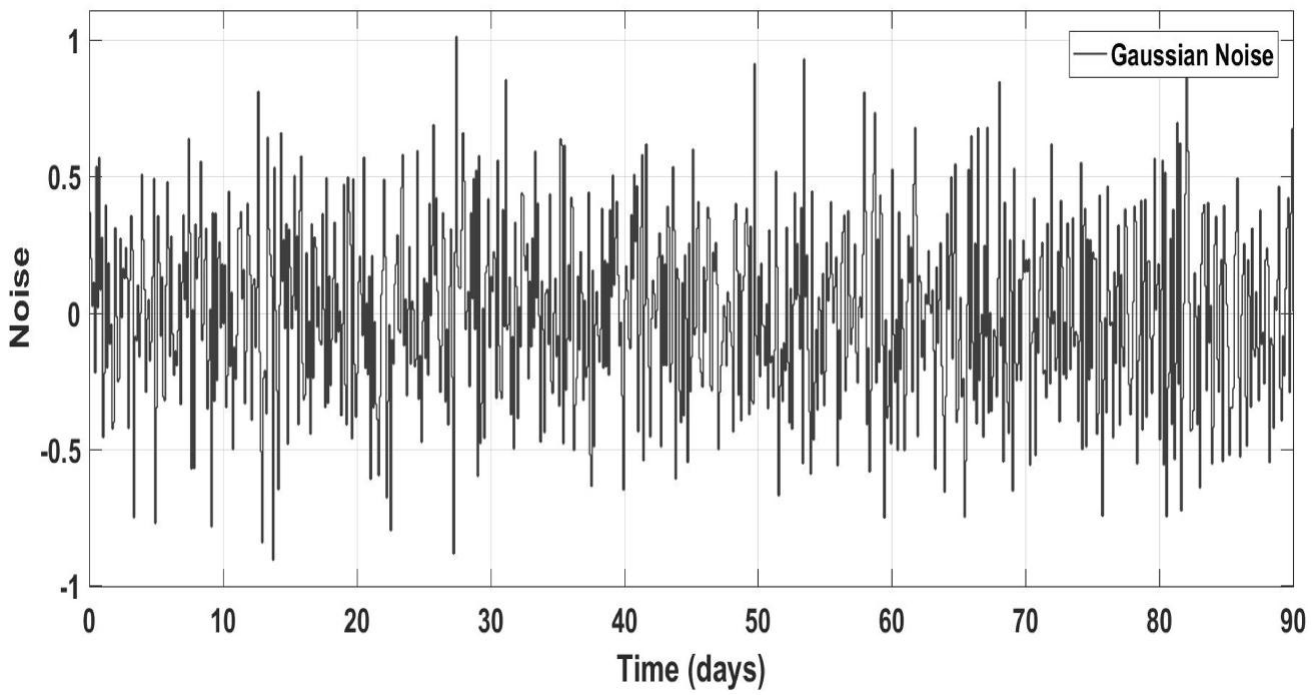
Gaussian noise

**FIGURE 27:**
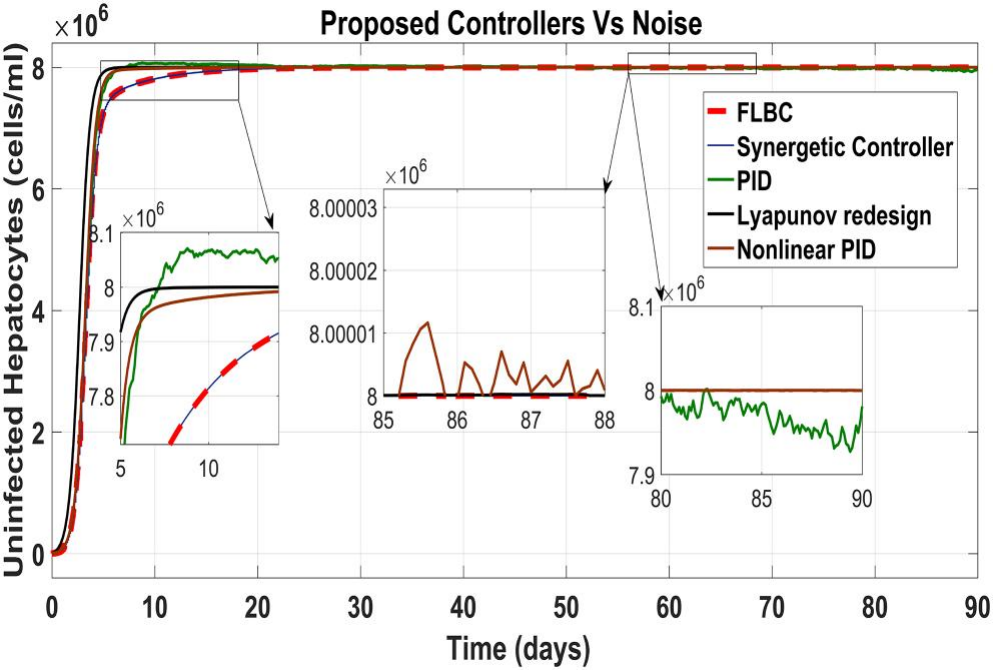
Behaviour of the uninfected hepatocytes

**FIGURE 28:**
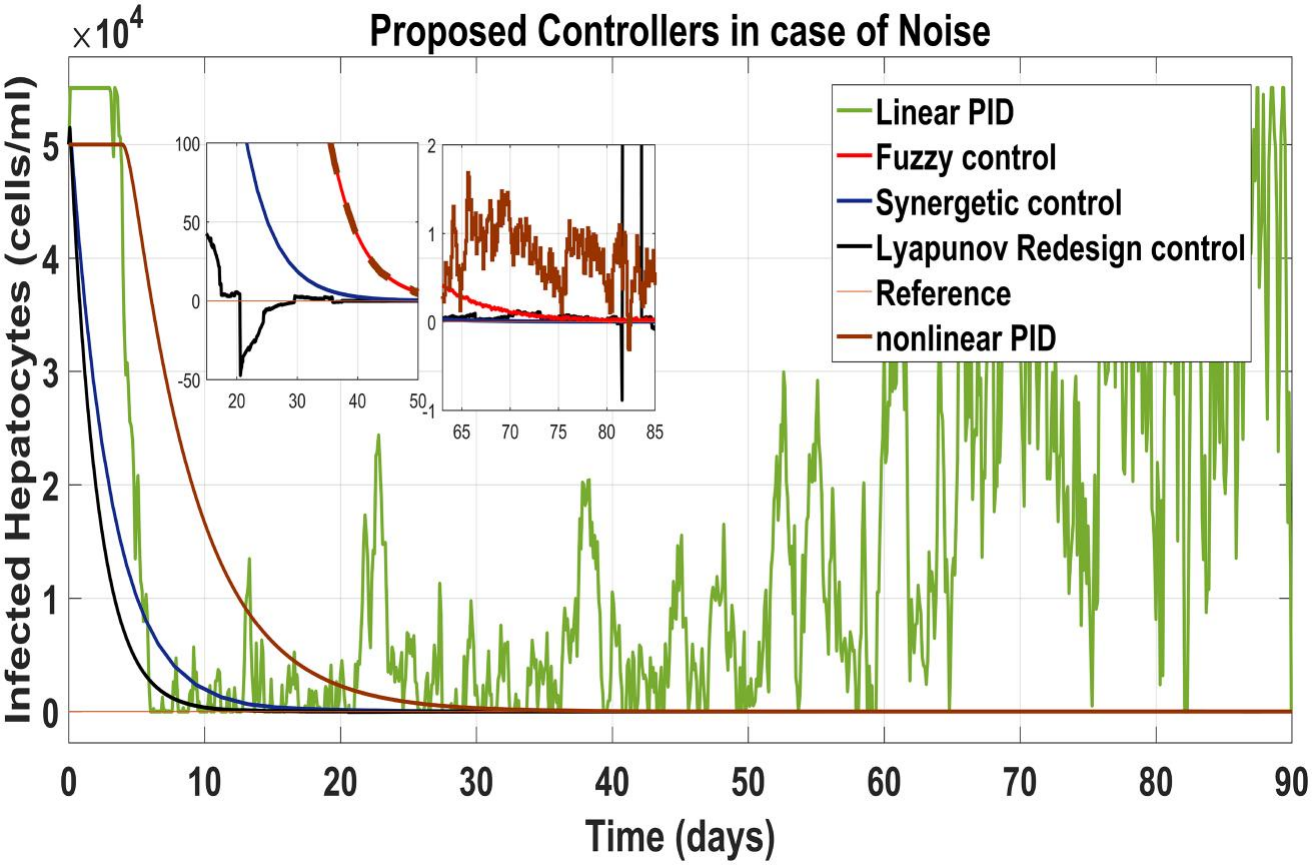
Behaviour of the infected hepatocytes

**FIGURE 29:**
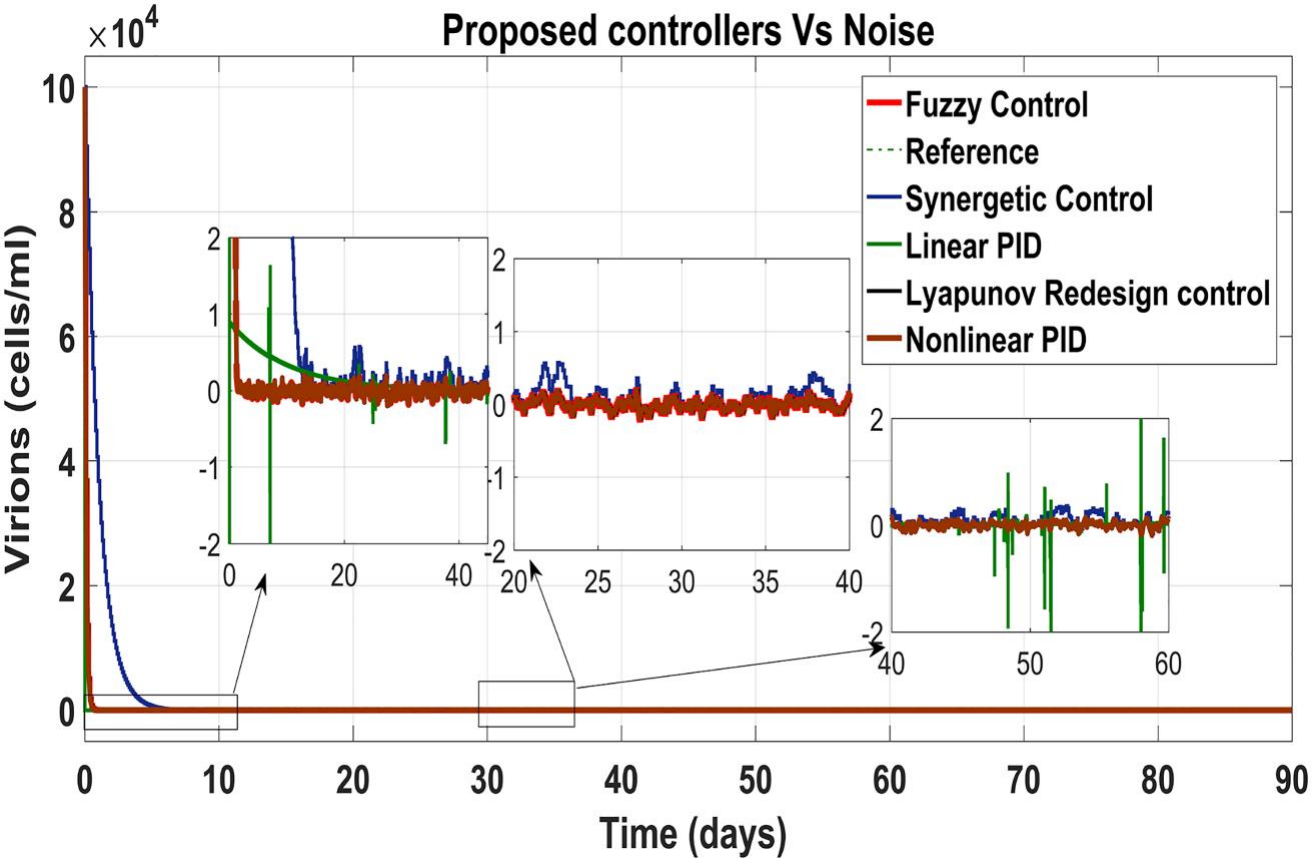
Behaviour of the virions

It shows that all proposed non‐linear controllers can tolerate such noise levels and track uninfected hepatocytes, infected hepatocytes and virions' state to their desired reference values with acceptable performance. Only the linear PID controller is unable to cope the disturbance and displays a very noisy output. The Lyapunov Redesign controller does have an undershoot, overshoot and disturbance in infected hepatocytes as shown in Figure 28. The FLBC and non‐linear PID show negligible SSE in the infected hepatocyte and uninfected hepatocyte states, respectively. Again, the results are more effective with the Synergetic controller having no SSE, ripples, overshoot/undershoot and fast convergence of all states.

## 6 CONCLUSION

Non‐linear controllers based on the non‐linear PID, Lyapunov Redesign control, Synergetic control and Fuzzy Logic have been proposed for the suppression of the infected hepatocytes and virions by using combined anti‐viral drugs peg‐IFN‐*α* and ribavirin as control inputs. Control input laws have been designed for reducing and blocking the infected hepatocytes and virions to their reference values. Drug efficacy limitations have also been taken into account. Simulation results in the MATLAB/Simulink show that the concentration of virions and infected hepatocytes can achieve reference values after approx 30 and 55 days, respectively. Also, the treatment strategy using a combination of anti‐viral drugs peg‐IFN‐*α* and ribavirin with the proposed model was observed depicting the infected hepatocytes and virions to reduce at a faster rate. As a result, the uninfected hepatocytes increase automatically and approach to their maximum limit *T*_max_. Non‐linear controllers have been compared with each other as well as with the linear PID on the basis of the transient response, settling time, SSE, ripples and undershoots/overshoots. It has been observed that the Synergetic controller performs better for blockage and reduction of infected hepatocytes and virions than the other proposed controllers and linear PID in the presence of additive noise. This work can be further extended by including the adaptation to reduce the un‐modelled uncertainties. Other non‐linear controllers such as SMC can also be implemented for robustness and quick convergence of the HCV system.
